# Mass spectrometry‐based proteomics analysis of human globus pallidus from progressive supranuclear palsy patients discovers multiple disease pathways

**DOI:** 10.1002/ctm2.1076

**Published:** 2022-11-10

**Authors:** Yura Jang, Thujitha Thuraisamy, Javier Redding‐Ochoa, Olga Pletnikova, Juan C. Troncoso, Liana S. Rosenthal, Ted M. Dawson, Alexander Y. Pantelyat, Chan Hyun Na

**Affiliations:** ^1^ Neuroregeneration and Stem Cell Programs Institute for Cell Engineering Johns Hopkins University School of Medicine Baltimore Maryland USA; ^2^ Department of Neurology Johns Hopkins University School of Medicine Baltimore Maryland USA; ^3^ Department of Pathology Johns Hopkins University School of Medicine Baltimore Maryland USA; ^4^ Department of Pathology and Anatomical Sciences Jacobs School of Medicine and Biomedical Sciences University at Buffalo Buffalo NY USA; ^5^ Solomon H. Snyder Department of Neuroscience Johns Hopkins University School of Medicine Baltimore Maryland USA; ^6^ Department of Pharmacology and Molecular Sciences Johns Hopkins University School of Medicine Baltimore Maryland USA

**Keywords:** electron transport chain complex, globus pallidus, mass spectrometry, progressive supranuclear palsy, proteomics, PSP

## Abstract

**Background:**

Progressive supranuclear palsy (PSP) is a neurodegenerative disorder clinically characterized by progressive postural instability, supranuclear gaze palsy, parkinsonism, and cognitive decline caused by degeneration in specific areas of the brain including globus pallidus (GP), substantia nigra, and subthalamic nucleus. However, the pathogenetic mechanism of PSP remains unclear to date.Unbiased global proteome analysis of patients' brain samples is an important step toward understanding PSP pathogenesis, as proteins serve as workhorses and building blocks of the cell.

**Methods:**

In this study, we conducted unbiased mass spectrometry‐based global proteome analysis of GP samples from 15 PSP patients, 15 Parkinson disease (PD) patients, and 15 healthy control (HC) individuals. To analyze 45 samples, we conducted 5 batches of 11‐plex isobaric tandem mass tag (TMT)‐based multiplexing experiments. The identified proteins were subjected to statistical analysis, such as a permutation‐based statistical analysis in the significance analysis of microarray (SAM) method and bootstrap receiver operating characteristic curve (ROC)‐based statistical analysis. Subsequently, we conducted bioinformatics analyses using gene set enrichment analysis, Search Tool for the Retrieval of Interacting Genes/Proteins (STRING) protein‐protein interaction (PPI) analysis, and weighted gene co‐expression network analysis (WGCNA).

**Results:**

We have identified 10,231 proteins with ∼1,000 differentially expressed proteins. The gene set enrichment analysis results showed that the PD pathway was the most highly enriched, followed by pathways for oxidative phosphorylation, Alzheimer disease, Huntington disease, and non‐alcoholic fatty liver disease (NAFLD) when PSP was compared to HC or PD. Most of the proteins enriched in the gene set enrichment analysis were mitochondrial proteins such as cytochrome c oxidase, NADH dehydrogenase, acyl carrier protein, succinate dehydrogenase, ADP/ATP translocase, cytochrome b‐c1 complex, and/or ATP synthase. Strikingly, all of the enriched mitochondrial proteins in the PD pathway were downregulated in PSP compared to both HC and PD. The subsequent STRING PPI analysis and the WGCNA further supported that the mitochondrial proteins were the most highly enriched in PSP.

**Conclusion:**

Our study showed that the mitochondrial respiratory electron transport chain complex was the key proteins that were dysregulated in GP of PSP, suggesting that the mitochondrial respiratory electron transport chain complex could potentially be involved in the pathogenesis of PSP. This is the first global proteome analysis of human GP from PSP patients, and this study paves the way to understanding the mechanistic pathogenesis of PSP.

## Graphical abstract

Proteome analysis of the globus pallidus from progressive supranuclear palsy, Parkinson's disease, and control individuals identified >10,000 proteins. Parkinson's disease, oxidative phosphorylation, and Alzheimer's disease pathways were the most enriched ones. The mitochondrial electron transport chain proteins were the key molecules downregulated in the globus pallidus of progressive supranuclear palsy patients

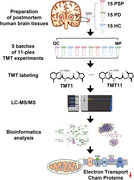

## 1 INTRODUCTION

Progressive supranuclear palsy (PSP) is a neurodegenerative disease clinically characterised by progressive parkinsonism, postural instability, vertical saccade slowing, supranuclear gaze palsy, and cognitive decline.^1^ PSP affects movement, gait, balance, speech, swallowing, vision, eye movement, mood, behaviour, and cognition.^2^, ^3^ The estimated prevalence of PSP is about five to six per 100,000 worldwide, and symptoms typically begin after the age of 60.^4^, ^5^ The disease is induced by the gradual degeneration of cells in specific areas of the brain, including the globus pallidus (GP), substantia nigra, subthalamic nucleus, and frontal lobes.^2^, ^6^, ^7^ The pathological hallmarks of PSP include the accumulation of four‐repeat tau proteins in neurofibrillary tangles, neuropil threads, and tau‐positive astrocytes.^1^ The underlying mechanisms of pathogenesis in PSP remain unclear. PSP is generally considered sporadic, but rare familial clusters have also been reported, and more than 10 genes with known mutations linked to PSP have been reported.^8^ The most studied gene in PSP is the microtubule‐associated protein tau (MAPT), which is expressed and regulated by alternative splicing in the human brain.^9^ MAPT H1 haplotype homozygosity significantly predisposes to PSP, and MAPT mutations cause familial PSP with monogenic autosomal dominant inheritance.^10^ Mutations in leucine‐rich repeat kinase 2 (LRRK2), which is known as one of the most common genetic causes of Parkinson's disease (PD), are also suggested as a cause of PSP, although more association studies are required.^8^, ^11^, ^12^, ^13^ Mutations of dynactin subunit 1 (DCTN1), which is one of the largest subunits in the dynactin family and is involved in cellular functions such as cell division and transport, were observed in patients with a clinical phenotype of PSP.^14^ Other genes with potential links to PSP include bassoon (BSN), chromosome 9 open reading frame 72 (C9orf72), eukaryotic translation initiation factor 2‐alpha kinase 3 (EIF2AK3), progranulin (GRN), myelin‐associated oligodendrocyte basic protein (MOBP), Niemann–Pick disease type C1 (NPC1), parkin (PARK2), Syntaxin 6 (STX6), TANK‐binding kinase 1 (TBK1), transactivation response element DNA‐binding protein (TARDBP), and several others.^8^, ^15^

Currently, there are no disease‐modifying treatments for PSP.^15^, ^16^ Symptoms of PSP are managed with medications for the treatment of other neurodegenerative diseases such as PD and AD, but effectiveness is limited.^6^, ^7^, ^16^, ^17^ To develop effective treatment of PSP, a deeper understanding of its pathogenetic mechanisms is essential. As such, it is crucial to identify proteins and relevant biological pathways involved in PSP pathogenesis. Since the advent of the mass spectrometry‐based proteomics approach, this method has been considered the gold standard for protein identification and measurement. Therefore, mass spectrometry‐based proteomic analysis of the human brain from PSP patients is essential to understand the pathogenesis of this disease. Nevertheless, no in‐depth global proteome data acquired from the brains of PSP patients is available to date. In this study, we conducted mass spectrometry‐based proteome analysis of GP from 15 PSP patients, 15 PD patients, and 15 healthy control (HC) individuals. To analyse and compare these 45 samples, we employed the 11‐plex isobaric tandem mass tag (TMT)‐based quantitative proteomics technology in which samples can be multiplexed up to 11 samples. To our knowledge, this study is the first in‐depth global proteome analysis of the GP from PSP patients. The proteins and relevant signalling pathways discovered in this study provide a foundation for unravelling the pathogenetic mechanisms of PSP.

## 2 METHODS

### 2.1 Collection of GP samples

We utilised GP samples from 15 PSP patients, 15 PD patients, and 15 HC individuals. GP was selected as a well‐defined basal ganglia region known to be affected by PSP pathology.^18^ The GP samples were collected from the Brain Resource Center at the Johns Hopkins University School of Medicine. The clinical information for the used samples is provided in Table 1 and Table S1. The inclusion criteria for PSP are patients with neuropathologic changes fulfilling PSP diagnostic criteria,^19^ age > 50 years, males and females, and any race. The exclusion criteria for PSP include patients with any significant neurodegenerative or vascular comorbidity.

**TABLE 1:** Demographics of GP samples from PSP patients, HC individuals, and PD patients used in this study

No.	Group	Age	Sex	Race	PMD (h)	CERAD	Braak stage	Diagnosis
1	PSP	77	F	W	2	0	3	PSP
2	PSP	65	F	W	4	0	3	PSP
3	PSP	76	F	W	NA	0	2	PSP
4	PSP	72	F	W	15	0	4	PSP
5	PSP	79	M	B	12	0	3	PSP
6	PSP	85	M	W	7	0	4	PSP
7	PSP	87	F	W	17	0	4	PSP
8	PSP	74	F	W	20	0	0	PSP, cerebrovascular disease not contributing
9	PSP	66	M	W	9.5	0	4	PSP, cerebrovascular disease not contributing
10	PSP	65	M	W	8	0	2	PSP
11	PSP	70	M	W	6	B	0	PSP, cerebrovascular disease not contributing
12	PSP	86	F	W	18.8	0	4	PSP, cerebrovascular disease not contributing
13	PSP	74	M	W	18	0	2	PSP
14	PSP	70	M	W	7.5	0	0	PSP, cerebrovascular disease not contributing
15	PSP	64	M	W	8.5	0	0	PSP
16	HC	80	F	W	6	0	0	Control
17	HC	86	F	B	6	A	2	Control
18	HC	66	M	W	12	0	3	Control
19	HC	70	M	W	0	NA	NA	Control
20	HC	80	F	W	8	0	0	Control
21	HC	74	M	W	4	0	2	Control
22	HC	79	M	W	16	0	2	Control
23	HC	87	F	W	7	0	2	Control, cerebrovascular disease not contributing
24	HC	89	M	W	9	0	2	Control, lacunar infarcts
25	HC	71	M	W	16	0	0	Control, lacunar infarct, cerebrovascular disease not contributing
26	HC	68	F	W	12	0	2	Control
27	HC	94	M	W	15	0	2	Control, asymptomatic Lewy bodies in SN and ERC
28	HC	90	F	B	22	A	3	Control
29	HC	77	M	W	10	0	4	Control, leukostasis from AML
30	HC	88	F	W	9	0	2	Control, CVD not contributing
31	PD	60	M	W	15.5	0	3	PD with dementia
32	PD	71	M	W	24	0	2	PD with dementia
33	PD	83	M	W	5	0	4	PD with dementia, neuro. degen, occipital infarct
34	PD	84	F	W	11	0	4	PD with dementia
35	PD	65	M	W	21	0	2	PD with dementia
36	PD	60	F	W	18	0	4	PD with dementia, cerebrovascular disease not contributing
37	PD	79	M	W	21	0	4	PD with dementia, neurofibrillary degeneration
38	PD	72	M	W	15	0	0	PD
39	PD	65	M	W	6	0	0	PD, cerebrovascular disease not contributing
40	PD	95	F	W	14	0	4	PD, neurofibrillary degeneration, cerebrovascular disease not contributing
41	PD	61	F	W	26	0	0	PD, juvenile PD, familial PD
42	PD	75	F	W	‐	0	2	PD, PART
43	PD	74	F	W	9.3	0	3	PD with dementia, part
44	PD	66	F	W	9	0	2	PD, PART
45	PD	92	F	W	17	0	2	PD

Abbreviations: AML, acute myeloid leukemia; B, Black; CERAD, Consortium to Establish a Registry for Alzheimer's Disease; CVD, cerebrovascular disease; ERC, entorhinal cortex; F, Female; HC, healthy control; M, Male; NA, not available; PART, primary age‐related tauopathy; PD, Parkinson's disease; PMD, post‐mortem delay; PSP, progressive supranuclear palsy; SN, substantia nigra; W, White.

### 2.2 Sample preparation and enzyme digestion of proteins from GP samples

All GP samples were prepared by sonicating (Branson sonifier 250, ultrasonics, Danbury, USA) in 8 M urea and 50 mM triethylammonium bicarbonate (TEAB) on ice. The protein amount of each sample was quantified by the bicinchoninic acid (BCA) protein assay (Pierce; Rockford, IL, USA). The 45 GP samples were divided into five batches to be analysed using 11‐plex TMT. Each batch included one master pool (MP) and one quality control (QC) sample. The MP and QC samples were prepared by combining an equal amount of proteins from all GP samples. The sample order for TMT labelling was randomised to minimise the effect of the TMT channel. The MP sample was added to the 11^th^ channel of each TMT experimental batch to normalise the data from multiple TMT experimental batches. The QC samples for verification of technical and biological variations between the batches were divided and placed in a channel in each batch before reduction and alkylation. For the reduction and alkylation of the proteins, 10 mM tris (2‐carboxyethyl) phosphine hydrochloride (TCEP) and 40 mM chloroacetamide (CAA) were added to the samples and then incubated for 1 h at room temperature (RT, 22°C to 25°C). Proteins were digested by LysC (Lysyl endopeptidase mass spectrometry grade; Fujifilm Wako Pure Chemical Industries Co., Ltd., Osaka, Japan) in a ratio of 1:100 for 3 h at 37°C, and then further digested by trypsin (sequencing grade modified trypsin; Promega, Fitchburg, WI, USA) in a ratio of 1:50 at 37°C overnight (for over 18 h) after diluting the concentration of urea from 8 M to 2 M by adding three volumes of 50 mM TEAB. The samples were acidified with 1% trifluoroacetic acid (TFA) to the final concentration and desalted with C18 Stage‐Tips (3M Empore^TM^; 3M, St. Paul, MN, USA). The eluted solution containing peptides was vacuum‐dried using a Savant SPD121P SpeedVac concentrator (Thermo Scientific, Waltham, MA, USA) and then stored at −80°C before use.^20^, ^21^

### 2.3 TMT labelling on the peptide samples and bRPLC fractionation

The digested peptides from GP samples were labelled using 11‐plex TMT reagents to perform TMT‐based quantitative mass spectrometry according to the manufacturer's instructions (Thermo Fisher Scientific, MA, USA). The MP sample was prepared in one tube and labelled by the 131C channel and split into five batches. The PSP, PD, HC, and QC samples were labelled with the rest of the channels. All TMT labelling reactions were performed for 1 h at RT and then quenched with 1/10 volume of 1 M Tris‐HCl (pH 8.0). The samples of each batch were pooled and subjected to prefractionation using basic pH reversed‐phase liquid chromatography (bRPLC) on an Agilent 1260 HPLC system (Agilent Technologies, Santa Clara, CA, USA). The TMT‐labelled peptides were reconstituted in solvent A (10 mM TEAB, pH 8.5) and loaded onto a C18 column (Agilent 300 Extend‐C18 column, 5 μm, 4.6 mm × 250 mm, Agilent Technologies). The loaded peptides were separated over the gradient of solvent B (10 mM TEAB in 90% acetonitrile (ACN), pH 8.5) at a flow rate of 0.3 mL/min. A total of 96 fractions collected over 97 min (the total run time of 150 min) were concatenated into 24 fractions. The concatenated samples were dried in a SpeedVac.^20^, ^21^, ^22^

### 2.4 LC‐MS/MS analysis

The prepared peptide samples were trapped onto an Acclaim™ PepMap™ 100 LC C18 NanoViper trap column (100 μm × 2 cm, packed with 5‐μm C18 particles, Thermo Scientific) at a flow rate of 8 μL/min and resolved on an EASY‐Spray™ analytical column (75 μm × 50 cm, packed with 2‐μm C18 particles, Thermo Scientific) at a flow rate of 0.3 μL/min using an Ultimate3000 RSLCnano nanoflow liquid chromatography system (Thermo Fisher Scientific, MA, USA) that was coupled with an Orbitrap Fusion Lumos Tribrid Mass Spectrometer. The peptide separation was conducted by increasing the gradient of solvent B (0.1% FA in 95% ACN) from 8% to 28% over 90 min. An EASY‐Spray ion source was operated at 2.4 kV. The data acquisition for the peptides injected into the mass spectrometer was conducted in data‐dependent acquisition (DDA) mode. The MS1 scan range was set to *m/z* 300 to 1,800 with a 3‐sec per cycle of the “top speed” setting. The mass resolutions for MS1 and MS2 were 120,000 and 50,000 at an *m/z* of 200, respectively. Maximum ion injection times for MS1 and MS2 were 50 and 100 milliseconds, respectively. The automatic gain controls for MS1 and MS2 were 1 and 0.05 million ions, respectively. The higher‐energy collisional dissociation (HCD) value was set to 35%. The precursor isolation window was set to *m/z* 1.5 with an *m/z* 0.4 offset. Dynamic exclusion was set to 30 s with 7 ppm of the mass window. Single‐charged ions were rejected. Internal calibration was conducted using the lock mass option (*m/z* 445.1200025) from ambient air.^20^, ^21^, ^22^

### 2.5 Data analysis

The data analysis was conducted as described in Khan et al. with some modifications as follows^21^: The version of Proteome Discoverer was 2.2.0.388. The UniProt database (released in May 2018) used in this study included both Swiss‐Prot and TrEMBL. The minimum peptide length was set to six amino acids. The MS order for the protein quantification was set to MS2. Reporter ion abundance was calculated based on the signal‐to‐noise (S/N) ratio. The average reporter ion S/N threshold and co‐isolation threshold were set to 50% and 30%, respectively.

### 2.6 Western blot assay

The GP tissues were heated at 95°C for 5 min and sonicated in RIPA lysis buffer (150 mM NaCl, 1% NP‐40, 25 mM Tris‐HCl pH 7.6, 0.1% sodium dodecyl sulfate, and 1% sodium deoxycholate) supplemented with an EDTA‐free protease inhibitor cocktail (Roche, Basel, Switzerland). Subsequently, the lysed samples were centrifuged at 16,000 × g at 4°C for 5 min. Protein quantification of supernatant from each sample was performed using the BCA protein assay (Pierce; Rockford, IL, USA). The samples were added with 4X Laemmli buffer (BIO‐RAD, Hercules, CA, USA) containing 10% 2‐mercaptoethanol and heated at 70°C for 10 min. The proteins were then separated on Novex WedgeWell 4 to 20% Tris‐Glycine gels (ThermoFisher Scientific, MA, USA). Proteins were blotted onto a 0.2‐μm polyvinylidene difluoride (PVDF) membrane (BIO‐RAD) using wet transfer at 100 V for 1.5 h. Subsequently, the PVDF membranes were blocked in StartingBlock (PBS) Blocking Buffer (Thermo Scientific) at RT for 1 h. Blocking buffer was used to dilute the primary and secondary antibodies. The PVDF membranes were incubated at 4°C overnight with one of the following primary antibodies: anti‐NDUFB11 (1:500, Invitrogen, Waltham, MA, USA), anti‐UQCRH (1:200, Invitrogen), anti‐NDUFA4 (1:1,000, Thermo Scientific), and anti‐*β*‐actin (Invitrogen). The next day, the PVDF membranes were washed three times in Tris‐buffered saline with Tween 20 TBST (Cell Signaling Technology, Danvers, MA, USA). Each wash was performed at RT for 10 min. Subsequently, the PDVF membranes were incubated with anti‐rabbit (1:1,000, Cell Signaling Technology) IgG secondary antibody conjugated with horseradish peroxide (HRP) at RT for 1 h. Finally, the membranes were washed three times again under the same wash conditions mentioned above, followed by incubation of the membranes with SuperSignal West Pico PLUS substrate (Thermo Fisher Scientific, MA, USA) for chemiluminescent detection. The membranes were imaged using a western blot imaging system (Amersham Imager 600, GE Healthcare, Milwaukee, WI, USA). Densitometric analysis of the images was performed on ImageJ software (NIH) and *t*‐test statistical analysis was performed for the relative intensity of *β*‐actin using GraphPad Prism version 9.4.0 for Windows (San Diego, CA, USA).

### 2.7 Experimental design and statistical rationale

The total number of GP samples used in this study was 45, composed of 15 PSP patients, 15 PD patients, and 15 HC individuals. We conducted sample size analysis using the pwr package in R. When we wanted to detect proteins with 1.5‐fold differences between groups, the required minimum sample size was 9.4 when the significance level was 0.0001, power was 0.8, sigma was 0.208, and delta was 0.585 ( = log_2_ 1.5). This sigma value of 0.208 was derived from our in‐house TMT proteomics experiments. The significance level of 0.0001 was determined based on our previous studies. When we identified several thousand proteins, the majority of the proteins with a *p* value < 0.0001 showed a *q*‐value < 0.05. Based on this sample size analysis, we decided to use 15 samples per group. The statistical analysis of the mass spectrometry data was performed with the Perseus version 1.6.0.7 software package. The quality of mass spectrometry data was monitored by measuring coefficient variations (CV) of QC samples and the S/N ratios. The S/N ratios were calculated by dividing standard deviations (SD) of the samples by SDs of QCs. The protein abundance data from five batches of the TMT experiments was normalised by dividing the abundance data of the PSP/PD/HC samples and QCs by those of the MPs included in each batch, followed by dividing by the median values of each protein. The relative abundance values for each sample were log_2_‐transformed, followed by a *z*‐score transformation.^23^, ^24^ We removed proteins with one or more missing values across 45 samples. To further remove batch effects, an additional normalization was conducted with the ComBat package in R.^25^ Proteins with a *q*‐value of <0.05 were considered significant. The fold changes between the comparison groups were calculated by dividing the average abundance values of each protein from one group by the values of another group. According to our normality test using Shapiro–Wilk test in the dplyr package in R, the majority of the proteins showed normal distribution. Thus, *p* values between the comparison groups were calculated by the student's two‐sample *t*‐test. Since we are conducting multiple comparisons, we calculated a false discovery rate by comparing data with and without permutations between groups. The *q*‐values for the volcano plots were calculated by a permutation‐based FDR estimation in the significance analysis of microarrays (SAM) method, in which *P* values and fold‐changes were calculated before and after the permutation of samples from two groups.^26^ As an orthogonal method to increase the reliability of the selection for differentially expressed proteins between groups, we also used bootstrap receiver operating characteristic (ROC) curve‐based statistical analysis.^27^, ^28^, ^29^, ^30^ A bootstrap ROC analysis was carried out using the fbroc package in R. Sampling with replacement was repeated 1,000 times for the bootstrap ROC. The area under the curve (AUC) of a bootstrap ROC was computed for each sampling. The mean and SD values of AUCs from 1,000 bootstrap ROC were then calculated.^31^, ^32^ The *q*‐values of the bootstrap ROC‐based analysis data were calculated as follows: (1) The mean AUC values for non‐permuted and permuted data were sorted in descending order for proteins with mean AUCs > 0.5 and in ascending order for proteins with mean AUCs < 0.5; (2) the ratios of the protein numbers for the non‐permuted data to the protein numbers for the permuted data were calculated as lowering the cut‐off threshold, and the ratios were used as *q*‐values.

### 2.8 Pathway analysis

The differentially expressed proteins were used for the Kyoto Encyclopedia of Genes and Genomes (KEGG) pathway analysis embedded in DAVID version 6.8.^33^, ^34^ Interactome analysis was carried out by the STRING PPI database version 11.^35^, ^36^ The weighted gene co‐expression network analysis (WGCNA) was conducted using the R software package.^37^, ^38^

### 2.9 Data and software availability

The mass spectrometry data from this study have been deposited to the ProteomeXchange Consortium (https://www.proteomexchange.org) via PRIDE partner repository with the dataset identifier PXD031648 and project name “Mass spectrometry‐based proteomics analysis of human globus pallidus from progressive supranuclear palsy patients discovers multiple disease pathways.”

## 3 RESULTS

### 3.1 Quantitative proteome analysis for the GP samples

**FIGURE 1:**
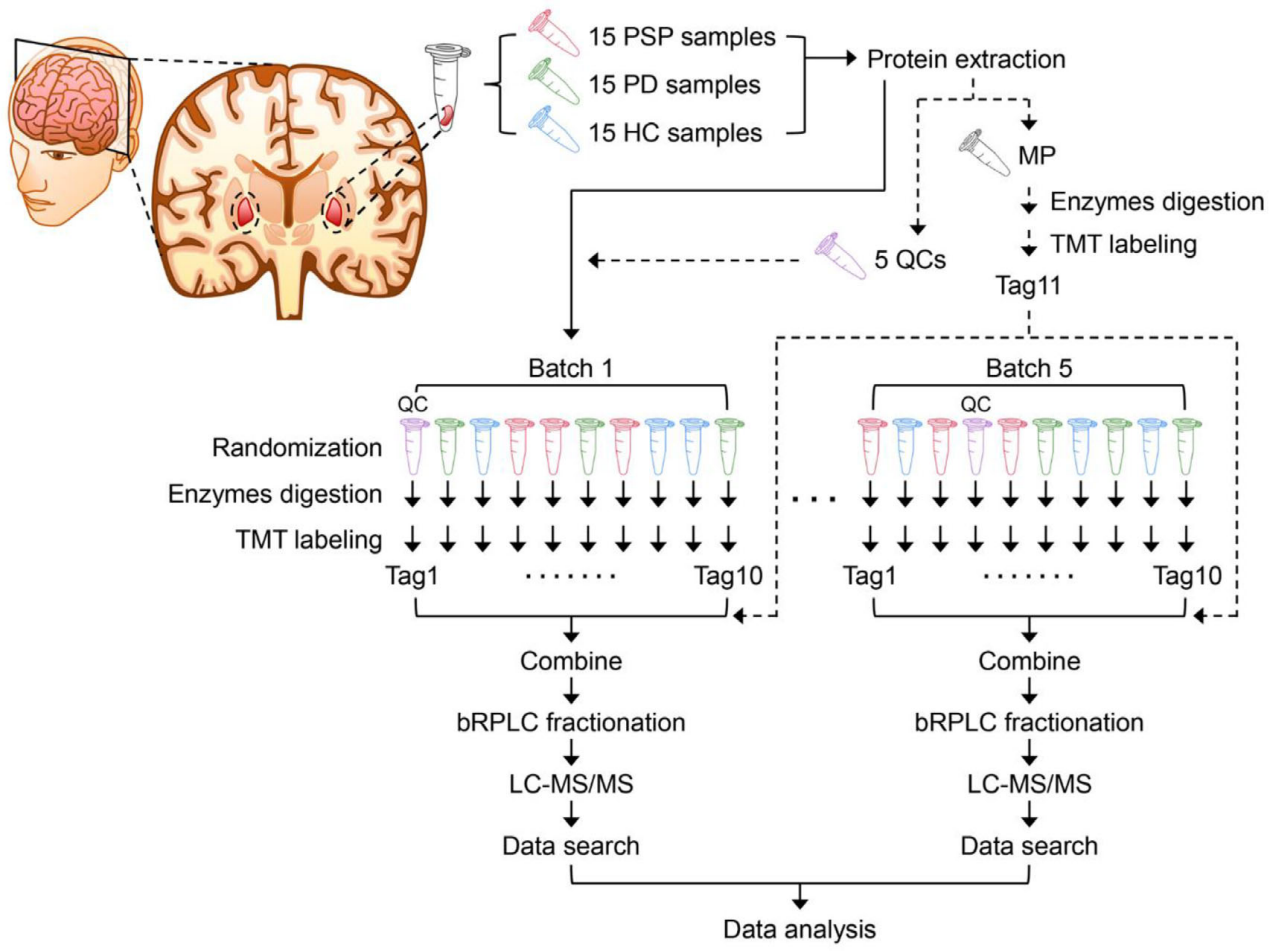
Experimental strategy for the proteomic study of the GP samples from PSP patients, PD patients, and HC individuals. Five batches of 11‐plex TMT experiments were conducted to analyse the proteome of human GP brain tissue samples from 15 PSP, 15 PD, and 15 HC. MP and QC samples were prepared by combining an equal amount of protein from all 45 GP samples. MP was added to each batch after labelling with Tag 11 in one tube. QC was split into five aliquots and processed in each batch separately. TMT tags for individual samples and QC were determined by randomization. The proteins were digested with Lys‐C and trypsin, followed by TMT labelling and prefractionation into 24 fractions prior to mass spectrometry analysis. Proteins were identified by conducting a database search of the acquired mass spectra.

**FIGURE 2:**
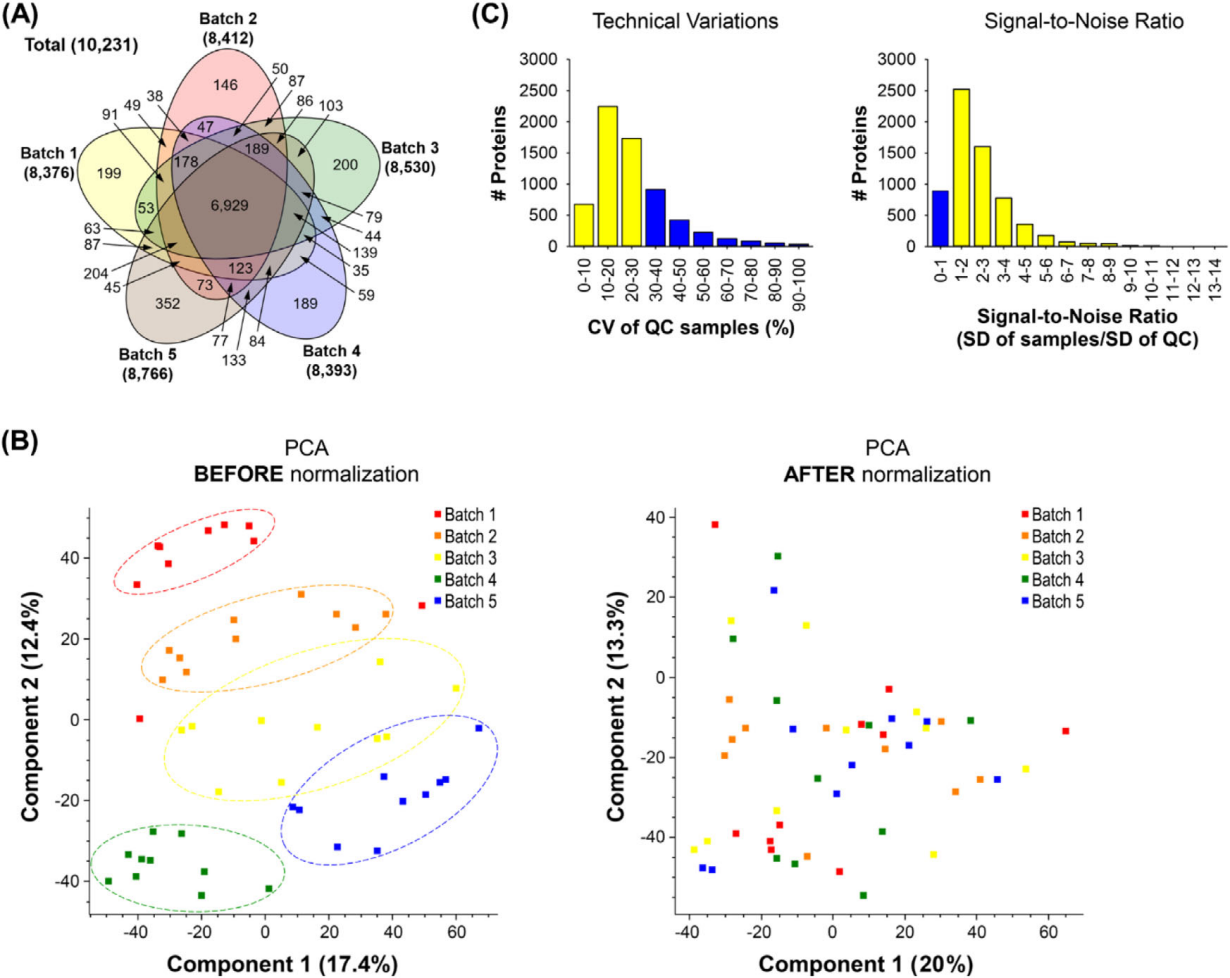
The evaluation of proteomic data quality from five batches of 11‐plex TMT experiments. (A) The number of Identified proteins in each batch is shown in a Venn diagram. (B) To minimise batch effects of five different 11‐plex TMT experiments, they were further normalised using the Combat package after normalizing each batch using MP. Forty‐five GP samples were shown on a 2D PCA plot to visualise potential batch effects before (left panel) and after (right panel) the normalization using the Combat package. (C) CV of QC samples and S/N ratio were calculated to evaluate data quality.

We conducted a quantitative proteome analysis of 45 GP samples from 15 PSP patients, 15 PD patients, and 15 HC individuals. For more accurate protein quantification, we exploited the 11‐plex TMT labelling method. For the analysis of 45 GP samples using 11‐plex TMT, we added MP to the 11^th^ channel of each TMT experimental batch to normalise data from multiple TMT experimental batches. A QC was placed in one of the remaining 10 channels of each TMT experimental batch to evaluate technical variations and the S/N ratio, as shown in Figure 1. The extracted proteins from human GP samples were digested with Lys‐C and trypsin, followed by TMT labelling and bRPLC fractionation. The fractionated peptides were then analysed on an LC‐MS/MS. In total, 5,223,768 of the MS/MS spectra were acquired, and 1,278,010 spectra were assigned to peptides, leading to the identification of 120,671 peptides and 10,231 proteins (Data S1). The numbers of proteins identified from each batch and all five batches in common were ∼8,500 and ∼6,900, respectively (Figure 2A). To compare protein abundances from five different batches, protein intensity values were normalised by the intensity values of the MP sample in each batch. Because the batch effect estimation by PCA analysis showed a residual batch effect, we conducted an additional normalisation using the ComBat package in R, and we observed that most of the residual batch effect was removed (Figure 2B). Subsequently, we accessed technical variations and the S/N ratio using the QC samples. More than 70% of proteins showed CV of <30%, and ∼90% of proteins showed S/N ratios > 1 (Figure 2C). These results suggest that our mass spectrometry analysis was successfully conducted with high precision.

### 3.2 Statistical analysis of the proteome data

**FIGURE 3:**
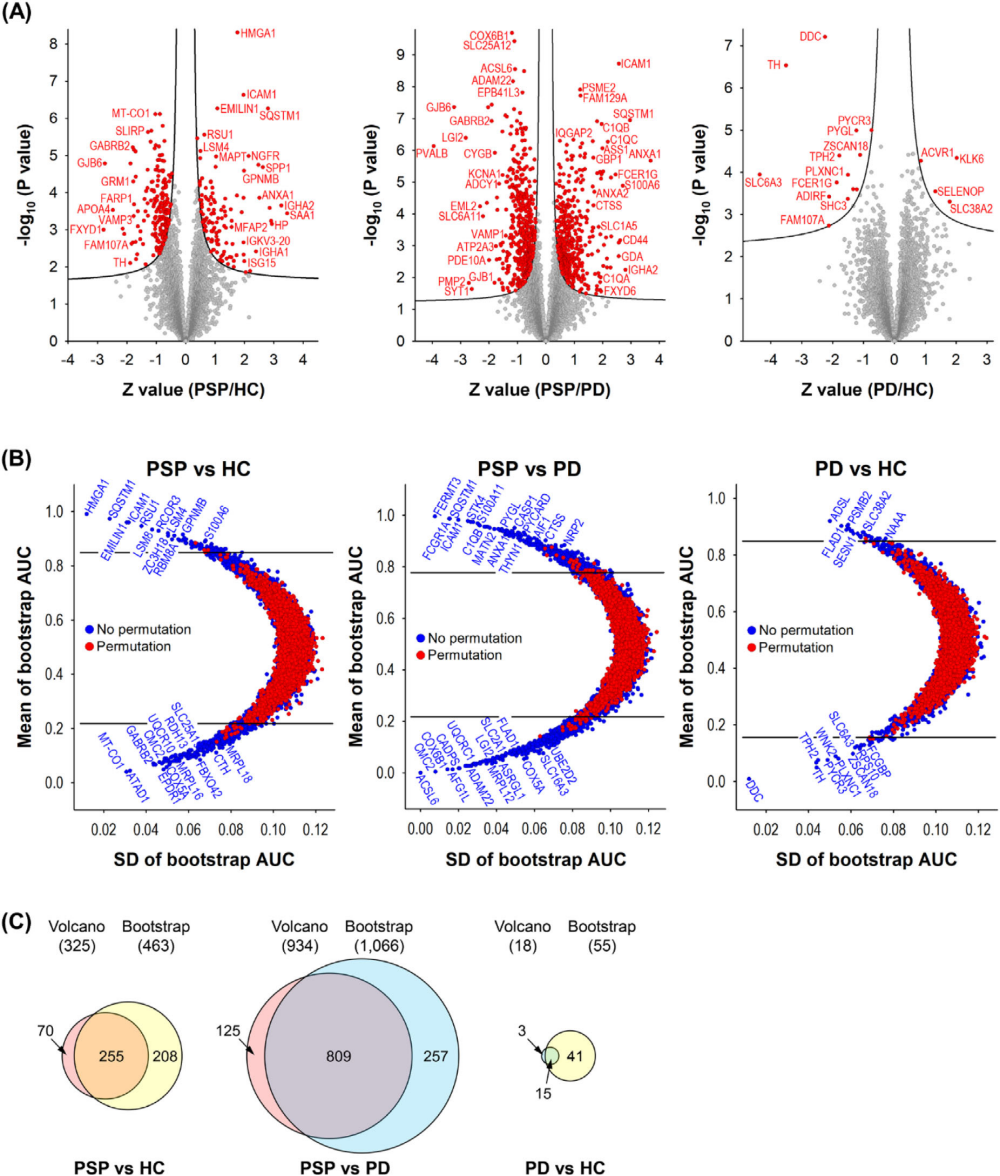
Volcano and bootstrap ROC plots of the GP proteins identified from PSP, PD, and HC. (A) The differences of GP protein abundances from 15 PSP, 15 PD, and 15 HC were depicted on volcano plots. The curved lines are the boundaries for a *q*‐value of 0.05. The proteins with the *q*‐value <0.05 are coloured in red. The proteins on the left and right sides of the *q*‐value line are down‐ and upregulated ones, respectively. (B) Bootstrap ROC analyses were conducted to estimate variations of resampling. To calculate *q*‐values, bootstrap ROC analyses after permutation of the comparison groups were conducted too. The differentially expressed proteins with *q*‐values < 0.05 are shown at the outside of the upper and lower horizontal lines. The proteins on the upper and lower side of the *q*‐value line are upregulated and downregulated in PSP compared to HC (left), in PSP compared to PD (middle), and in PD compared to HC (right), respectively. (C) The differentially expressed proteins overlapping between the volcano plot and the bootstrap ROC analysis of each comparison group are shown in the Venn diagrams.

To identify proteins with differential expression in GP from PSP patients compared to PD or HC individuals, statistical analyses of the proteome data were conducted using two different approaches: SAM‐ and bootstrap ROC‐based analyses. In the SAM‐based approach, the numbers of differentially expressed proteins in PSP versus HC, PSP versus PD, and PD versus HC were 325, 934, and 18, respectively (Figure 3A; Tables S2–S4; and Data S2). HMGA1, ICAM1, EMILIN1, SQSTM1, NGFR, SPP1, IGHA2, SAA1, and so on were among the most upregulated proteins, and MT‐CO1, SLIRP, GABRB2, GJB6, APOA4, FXYD1, and so on were among the most downregulated proteins in PSP compared to HC. ICAM1, PSME2, FAM129A, SQSTM1, ANXA1, FCER1G, S100A6, CD44, GDA, IGHA2, and so on were among the most upregulated proteins, and COX6B1, SLC25A12, ACSL6, ADAM22, GJB6, LGI2, PVALB, EML2, SLC6A11, PDE10A, and so on were among the most downregulated proteins in PSP compared to PD. ACVR1, KLK6, SELENOP, SLC38A2, and so on were among the most upregulated proteins and DDC, TH, TPH2, SLC6A3, FCER1G, ADIRF, and so on were among the most downregulated proteins in PD compared to HC (Figure 3A).

**FIGURE 4:**
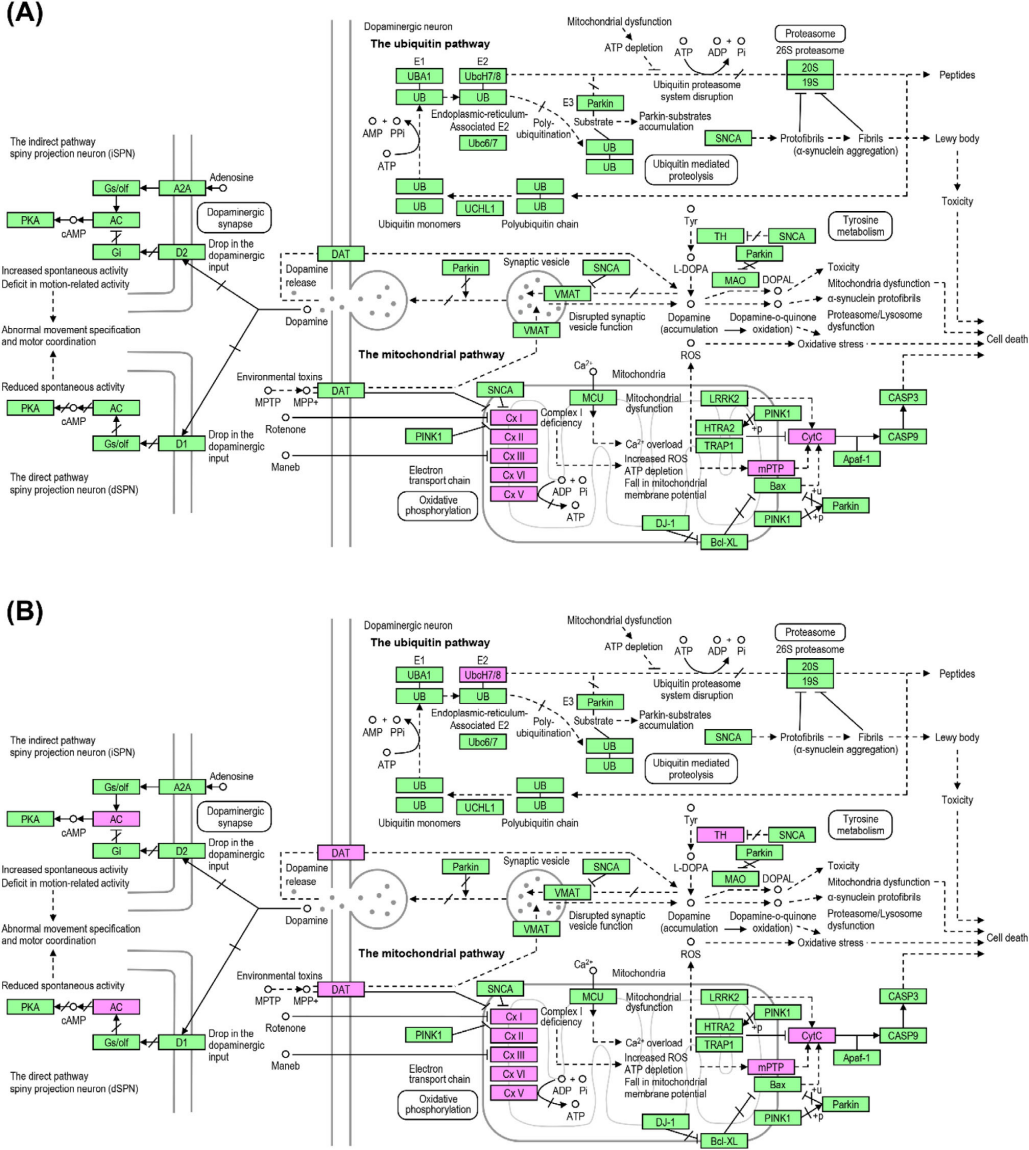
Parkinson's disease pathway map identified by the gene set enrichment analysis. The Parkinson's disease pathway map was selected by gene set enrichment analysis with differentially expressed proteins in the comparison between (A) PSP and HC and between (B) PSP and PD using the KEGG pathway database. The differentially expressed proteins identified in this study are coloured in magenta.

In the bootstrap ROC‐based approach, the numbers of differentially expressed proteins in PSP versus HC, PSP versus PD, and PD versus HC were 463, 1,066, and 55, respectively (Figure 4B; Tables S5–S7; and Data S2). HMGA1, SQSTM1, EMILIN1, ICAM1, RSU1, LSM8, RCOR3, ZC3H18, LSM4, and so on were among the most upregulated proteins, and MT‐CO1, ATAD1, GABRB2, EPDR1, CMC2, COX5A, UQCR10, MRPL16, and so on were among the most downregulated proteins in PSP compared to HC. FERMT3, FCGR1A, SQSTM1, ICAM1, STK4, S100A11, C1QB, MATN2, and so on were among the most upregulated proteins, and ACSL6, CMC2, COX6B1, AFG1L, CADPS, ADAM22, UQCRC1, MRPL12, LGI2, and so on were among the most downregulated proteins in PSP compared to PD. ADSL, FLAD1, PSMB2, SESN1, SLC38A2, NAAA, and so on were among the most upregulated proteins, and DDC, TH, TPH2, PYCR3, WNK2, PLXNC1, ZSCAN18, SLC6A3, RPS10, and so on were among the most downregulated proteins in PD compared to HC (Figure 3B).

When the differentially expressed proteins identified from the SAM‐based analysis were compared with those identified with bootstrap ROC analysis, 225, 809, and 15 proteins overlapped in PSP versus HC, PSP versus PD, and PD versus HC, respectively (Figure 3C). To minimise the number of differentially expressed proteins selected by type I error, we decided to use the differential proteins common to both of our analytic approaches for further pathway analysis.

### 3.3 Gene set enrichment analysis

To uncover dysregulated signalling pathways in the GP of PSP, gene set enrichment analysis was conducted using the KEGG pathway database embedded in DAVID bioinformatics resources (Data S3). When PSP was compared to HC, the PD pathway was the most enriched one, followed by oxidative phosphorylation, Alzheimer's disease, Huntington's disease, and non‐alcoholic fatty liver disease (NAFLD) pathways (Table 2). For the PD pathway, 25 proteins were enriched, and strikingly, the majority of the proteins enriched in the pathway were proteins related to mitochondrial functions such as electron transport (COX4I1, COX5A, COX6B1, COX7A2, CYCS, and NDUFA4), NADH dehydrogenases (NDUFA9, NDUFB11, NDUFB3, NDUFB4, NDUFB7, NDUFB8, NDUFC1, NDUFS2, NDUFS5, NDUFS7, and NDUFS8), succinate dehydrogenase (SDHC), ADP/ATP translocase (SLC25A4 and SLC25A5), and cytochrome b‐c1 complex proteins (UQCR10, UQCR11, UQCRB, UQCRH, and UQCRQ) (Figure 4A). The majority of proteins enriched in the four other pathways were also related to mitochondrial functions (Table S4). All of the mitochondria‐related proteins were downregulated in PSP compared to HC.

**TABLE 2:** Pathways enriched by gene set enriched pathways of differentially expressed proteins in PSP compared to HC. Differentially expressed proteins selected by both volcano plot and bootstrap ROC analysis were used for this analysis

Term	Count	^a^PH	%	*p* Value
Parkinson's disease	25	142	17.6	3.40E‐18
Oxidative phosphorylation	24	133	18.0	1.10E‐17
Alzheimer's disease	25	168	14.9	1.90E‐16
Huntington's disease	26	192	13.5	3.90E‐16
Non‐alcoholic fatty liver disease (NAFLD)	23	151	15.2	2.80E‐15
Ribosome	19	136	14.0	6.40E‐12
Metabolic pathways	42	1219	3.4	2.80E‐06
Cardiac muscle contraction	9	75	12.0	3.20E‐05
Glutamatergic synapse	7	114	6.1	1.20E‐02
Citrate cycle (TCA cycle)	4	30	13.3	1.30E‐02

^a^ PH, the total number of proteins in the pathway.

When PSP was compared to PD, the top five most enriched pathways were the same as the ones enriched in the comparison between PSP and HC, but the number of enriched proteins in each pathway was more than doubled (Table 3). For the PD pathway, 60 proteins were enriched and the majority of the proteins were related to mitochondrial functions, as was observed in the comparison between PSP and HC (Figure 4B). These mitochondrial proteins included ATP synthase (ATP5F1, ATP5H, ATP5J, and ATP5O) and acyl carrier protein (NDUFAB1) as well as the ones already observed in the comparison between PSP and HC, such as cytochrome c‐related proteins (COX4I1, COX5A, COX5B, COX6A1, COX6B1, COX6C, COX7A2, COX7A2L, COX7C, CYC1, CYCS, and NDUFA4), NADH dehydrogenases (NDUFA2, NDUFA3, NDUFA4, NDUFA7, NDUFA9, NDUFA10, NDUFA12, NDUFA13, NDUFAB1, NDUFB3, NDUFB4, NDUFB5, NDUFB6, NDUFB7, NDUFB8, NDUFB9, NDUFB10, NDUFB11, NDUFC1, NDUFC2, NDUFS1, NDUFS2, NDUFS3, NDUFS5, NDUFS7, NDUFS8, NDUFV1, and NDUFV3), succinate dehydrogenase (SDHA, SDHB, and SDHC), ADP/ATP translocase (SLC25A4, SLC25A5, and SLC25A6), and cytochrome b‐c1 complex proteins (UQCR11, UQCRB, UQCRC1, UQCRC2, UQCRFS1, UQCRH, and UQCRQ). The majority of proteins enriched in the four other pathways were also related to mitochondrial function, as observed in the comparison between PSP and HC (Table S4). All of the mitochondria‐related proteins were downregulated in PSP compared to HC. The gene set enrichment analysis results suggest that the downregulation of mitochondrial proteins is potentially linked to PSP pathogenesis.

**TABLE 3:** Pathways enriched by gene set enriched pathways of differentially expressed proteins in PSP compared to PD. Differentially expressed proteins selected by both volcano plot and bootstrap ROC analysis were used for this analysis

Term	Count	^a^PH	%	*p* Value
Parkinson's disease	60	142	42.3	3.80E‐38
Oxidative phosphorylation	55	133	41.4	2.70E‐34
Alzheimer's disease	58	168	34.5	3.10E‐31
Huntington's disease	59	192	30.7	1.00E‐28
Non‐alcoholic fatty liver disease (NAFLD)	49	151	32.5	6.30E‐25
Cardiac muscle contraction	24	75	32.0	4.40E‐12
Spliceosome	26	133	19.5	4.10E‐08
Metabolic pathways	109	1219	8.9	5.00E‐08
Pancreatic secretion	15	93	16.1	4.90E‐04
Salivary secretion	13	86	15.1	2.40E‐03

^a^ PH, the total number of proteins in the pathway.

### 3.4 Protein‐protein interaction analysis

**FIGURE 5:**
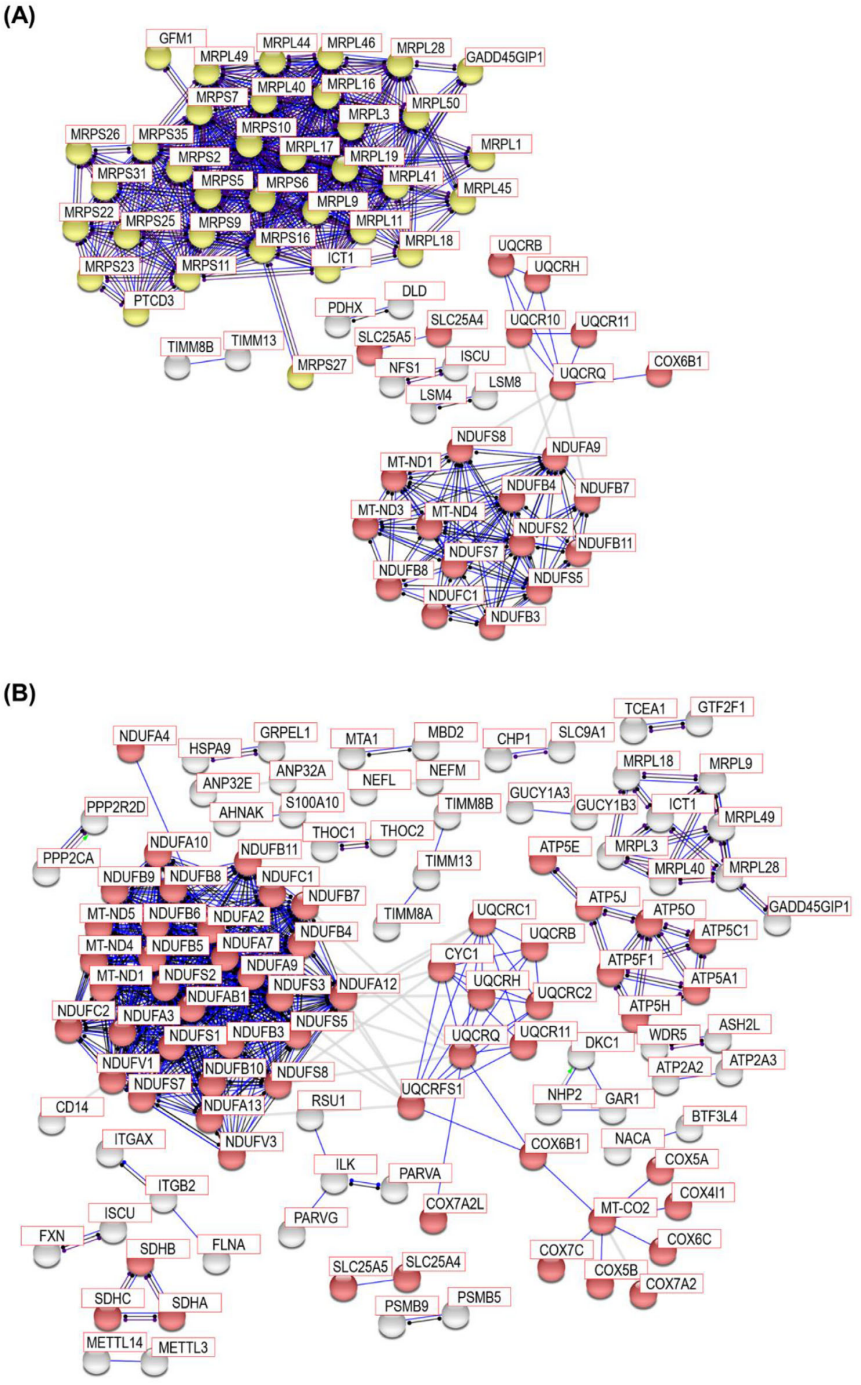
STRING PPI analysis for network connectivity of the differentially expressed proteins in PSP compared to HC and in PSP compared to PD. STRING PPI analyses were conducted to estimate the connectivity of the differentially expressed proteins (A) in PSP compared to HC and (B) in PSP compared to PD. In the comparison between PSP and HC, the network contains 250 nodes with 980 edges. Only experimental data was used for the active interaction source with 0.9 highest confidence threshold of a minimum required interaction score (average node degree: 7.84, average local clustering coefficient: 0.482, and PPI enrichment *p* value < 1.0 ×10^−16^). In the comparison between PSP and PD, the network contains 796 nodes with 503 edges. Only experimental data was used for the active interaction source with 0.9 highest confidence threshold of a minimum required interaction score (average node degree: 1.26, average local clustering coefficient: 0.14, and PPI enrichment *p* value < 1.0 ×10^−16^). The red and yellow nodes denote Parkinson's disease and mitochondria translation‐related proteins, respectively. The grey nodes do not belong to any enriched pathways.

The gene set enrichment analysis showed that most of the proteins enriched in the top five pathways were related to mitochondrial function. We reasoned that the analysis of differential proteins using an orthogonal approach would provide higher confidence in the relevance of mitochondrial proteins in PSP. For this, we conducted a PPI analysis of the differentially expressed proteins in the comparison groups using STRING PPI analysis. We used ‘Experiment’ alone as an active interaction source and a minimum required interaction score threshold of 0.9 (highest confidence). For the differentially expressed proteins in the comparison between PSP and HC, the STRING PPI analysis produced two highly connected clusters and one moderately connected cluster (Figure 5A). The most connected cluster was formed by mitochondrial ribosomal proteins (MRPs). The second and third most connected clusters were formed by the NADH dehydrogenases and cytochrome b‐c1 complex proteins. Reactome analysis embedded in STRING PPI also showed that all of the top four enriched pathways were related to mitochondrial translation (Table 4). For the differentially expressed proteins in the comparison between PSP and PD, STRING PPI analysis produced one highly connected and three moderately connected clusters (Figure 5B). All connected clusters were formed by proteins related to mitochondrial function, such as NADH dehydrogenase, ATP synthase, cytochrome b‐c1 complex with cytochrome c oxidase, and succinate dehydrogenase. Reactome analysis also showed that all of the top three enriched pathways were related to mitochondrial respiratory electron transport (Table 5). The interactome analysis results also suggest that mitochondrial proteins represent the main component of the differentially expressed proteins in PSP compared to HC and PD.

**TABLE 4:** Reactome analysis using the differentially expressed proteins in PSP compared to HC. Differentially expressed proteins selected by both volcano plot and bootstrap ROC analysis were used for this analysis

Pathway	Description	Count in gene set	False discovery rate
HSA‐5368287	Mitochondrial translation	39 of 94	4.26E‐40
HSA‐5419276	Mitochondrial translation termination	38 of 88	7.69E‐40
HSA‐5389840	Mitochondrial translation elongation	38 of 88	7.69E‐40
HSA‐5368286	Mitochondrial translation initiation	37 of 88	1.07E‐38
HSA‐1428517	The citric acid (TCA) cycle and respiratory electron transport	38 of 173	5.93E‐31
HSA‐163200	Respiratory electron transport, ATP synthesis by chemiosmotic coupling, and heat production by uncoupling proteins	32 of 123	6.31E‐28
HSA‐611105	Respiratory electron transport	30 of 100	9.74E‐28
HSA‐72766	Translation	40 of 288	4.92E‐26
HSA‐6799198	Complex I biogenesis	16 of 55	2.12E‐14
HSA‐1268020	Mitochondrial protein import	15 of 64	2.45E‐12

**TABLE 5:** Reactome analysis using the differentially expressed proteins in PSP compared to PD. Differentially expressed proteins selected by both volcano plot and bootstrap ROC analysis were used for this analysis

Pathway	Description	Count in gene set	False discovery rate
HSA‐163200	Respiratory electron transport, ATP synthesis by chemiosmotic coupling, and heat production by uncoupling proteins	71 of 123	8.51E‐47
HSA‐1428517	The citric acid (TCA) cycle and respiratory electron transport	77 of 173	6.98E‐45
HSA‐611105	Respiratory electron transport	63 of 100	1.90E‐43
HSA‐1430728	Metabolism	196 of 2032	1.65E‐26
HSA‐6799198	Complex I biogenesis	35 of 55	8.37E‐24
HSA‐1268020	Mitochondrial protein import	30 of 64	1.90E‐17
HSA‐9609507	Protein localization	31 of 122	5.43E‐12
HSA‐72203	Processing of Capped Intron‐Containing Pre‐mRNA	40 of 234	8.77E‐11
HSA‐72163	mRNA splicing ‐ Major pathway	32 of 178	5.18E‐09
HSA‐72172	mRNA splicing	32 of 186	1.26E‐08

### 3.5 Protein co‐expression network analysis

**FIGURE 6:**
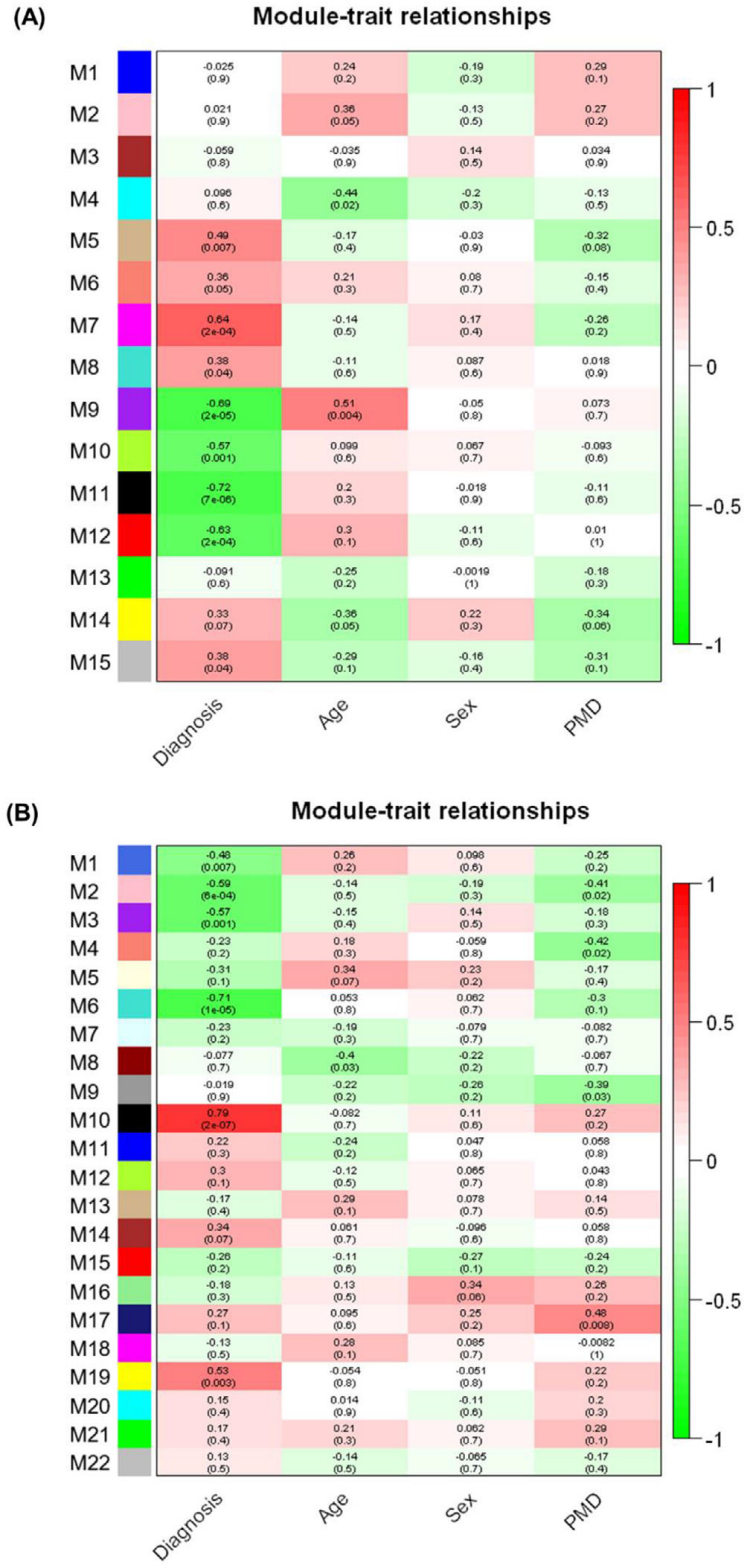
WGCNA of GP proteome data to investigate the module‐trait relationships. The module‐trait relationships of the GP proteome data using WGCNA were presented in the form of heatmaps. WGCNA was conducted with proteins identified from (A) PSP and HC or (B) PSP and PD. Each module is composed of a group of proteins with similar expression patterns. The relationships between modules and traits were calculated by calculating Pearson correlations between modules and traits. The correlation scores are displayed on the top of each box. Red and green colours represent positive and negative correlations, respectively. *p* Values for the significance between modules and traits were calculated and displayed on the bottom of each box in the parenthesis.

Both the gene set enrichment and the PPI analyses for the differentially expressed proteins in the GP of PSP patients suggested that mainly mitochondrial proteins were dysregulated. However, we still could not exclude the possibility that variables other than PSP pathology could contribute to the differential expression of mitochondrial proteins. For this, we conducted WGCNA, in which proteins with similar co‐expression patterns are identified, generating multiple modules that are composed of proteins with similar expression patterns. Subsequently, the correlations of the modules with various traits of the samples such as diagnosis, age, sex, and post‐mortem delay (PMD) are estimated (Table 1; Figure 6; and Figure S1). Since the PD, mitochondrial translation, and ribosome pathways were the ones most enriched in the gene set enrichment and the PPI analyses, we investigated whether the modules enriched with proteins belonging to these three pathways were correlated with variables other than disease diagnosis. First, we selected modules that showed correlations with the disease diagnosis trait and searched them for the modules that have proteins enriched with the three pathways among them.

When PSP and HC data were analysed using WGCNA, the M5, M7, M8, and M15 modules showed positive correlations with *p* values of <0.05, demonstrating that the proteins in these clusters have a tendency to have increased expression levels in PSP compared to HC. On the other hand, the M9, M10, M11, and M12 modules showed negative correlations with *p* values of <0.05, indicating that the proteins in these clusters have a tendency to have decreased expression levels in PSP compared to HC (Figure 6A). In the M12 module, the top five pathways selected by the gene set enrichment analysis were the same as those observed for differential proteins between PSP and HC, with the PD pathway being the most enriched (Table S8; Table 2; and Data S4). In the M11 module, the ribosome pathway was the most enriched (Table S7 and Data S4). When PSP and PD data were analysed using WGCNA, the M10 and M19 modules showed positive correlations with *p* values of <0.05, demonstrating that the proteins in these clusters have a tendency to have increased expression levels in PSP compared to PD. On the other hand, the M1, M2, M3, and M6 modules showed negative correlations with *p* values of <0.05, indicating that the proteins in these clusters have a tendency to have decreased expression levels in PSP compared to PD (Figure 6B). In the M6 module, the top five pathways selected by the gene set enrichment analysis were the same as those observed for differential proteins between PSP and PD, with the PD pathway as the most enriched (Table S9; Table 3; and Data S4).

**FIGURE 7:**
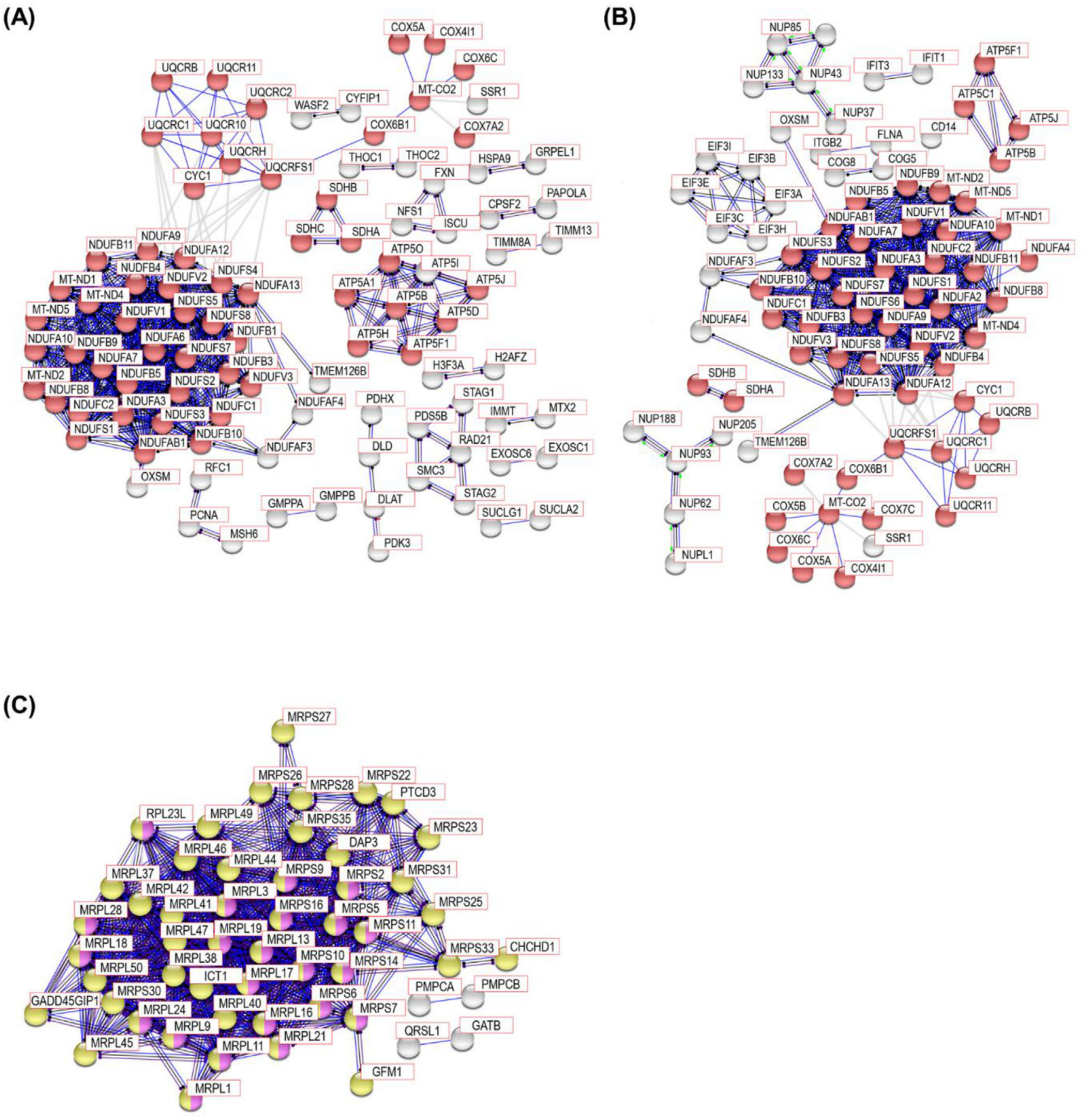
STRING PPI analysis for network connectivity of the modules from WGCNA enriched with Parkinson's disease, mitochondria translation, and ribosome pathways. STRING PPI analysis with the proteins in (A) the M12 module generated by WGCNA of PSP and HC, (B) the M6 module generated by WGCNA of PSP and PD, and (C) the M11 module generated by WGCNA of PSP and HC. In the M12 module from PSP and HC, the network contains 422 nodes with 501 edges. Only experimental data was used for the active interaction source with 0.9 highest confidence threshold of a minimum required interaction score (average node degree: 2.37, average local clustering coefficient: 0.193, and PPI enrichment *p* value <1.0 ×10^−16^). In the M6 module from PSP and PD, the network contains 997 nodes with 702 edges. Only experimental data was used for the active interaction source with 0.9 highest confidence threshold of a minimum required interaction score (average node degree: 1.41, average local clustering coefficient: 0.203, and PPI enrichment *p* value <1.0 ×10^−16^). In the M11 module from PSP and HC, the network contains 187 nodes with 542 edges. Only experimental data was used for the active interaction source with 0.9 highest confidence threshold of a minimum required interaction score (average node degree: 5.8, average local clustering coefficient: 0.219, and PPI enrichment *p* value <1.0 ×10^−16^). The red, yellow, and magenta nodes denote Parkinson's disease, mitochondria translation, and ribosome pathways, respectively. The grey nodes do not belong to any enriched pathways.

Subsequently, we conducted the PPI analysis with proteins in the M12 and M11 modules generated by WGCNA of PSP and HC and the M6 module generated by WGCNA of PSP and PD. The M12 module from PSP and HC and the M6 module from PSP and PD showed highly connected clusters for mitochondrial respiratory electron transport chain proteins such as NADH dehydrogenase, ATP synthase, cytochrome c oxidase, cytochrome b‐c1 complex, and succinate dehydrogenase (Figures 7A,B), and they were enriched in the PD pathway. The M11 module from PSP and HC showed highly connected clusters for MRPs and they were enriched in mitochondrial translation and ribosome pathways (Figure 7C). These WGCNA results showed that the M12 and M11 modules from PSP and HC and the M6 module from PSP and PD were enriched for mitochondria‐related proteins. However, none of the modules exhibited high correlations with variables other than the diagnostic group. This suggests that the mitochondria‐related proteins in this study are highly linked to PSP pathogenesis, although the causality of the mitochondria‐related proteins for PSP pathogenesis remains to be further investigated.

### 3.6 Validation of differentially expressed proteins in the mitochondrial electron transport chain

Since the bioinformatic analysis of the proteomics data acquired in this study revealed that mitochondrial electron transport chain proteins were key molecules differentially regulated in the GP of the PSP, we conducted Western blot experiments for the validation of proteins in the mitochondrial electron transport chain in the GP from an independent cohort composed of 4 PSP, 4 PD, and 4 HC individuals (Table S1). We selected five proteins (SDHC, SLC25A5, NDUFB11, UQCRH, and NDUFA4) in the electron transport chain for the validation experiments, but only three proteins (NDUFB11, UQCRH, and NDUFA4) were detectable by Western blot. Only NDUFA4 showed a decrease in PSP with statistical significance (Figure S2). These results are likely due to sample preparation differences between proteomics and Western blot experiments. Although quantifications by MS and Western blot did not confirm all three proteins, it is well‐known that mass spectrometry‐based quantification data often shows a poor correlation with Western blot‐based quantification.^39^, ^40^

## 4 DISCUSSION

In this study, we conducted mass spectrometry‐based proteome analysis of human GP brain tissue samples from 15 PSP patients, 15 PD patients, and 15 HC individuals using a TMT‐based multiplexing method, in which we identified ∼10,000 proteins and ∼120,000 peptides. To our best knowledge, this is the first in‐depth proteome analysis of the human GP region from PSP patients. In this study, we used two different methods for the selection of differentially expressed proteins. We identified 325, 934 and 18 differentially expressed proteins in the comparisons between PSP and HC, between PSP and PD, and between PD and HC, respectively, using SAM‐based statistical analysis. On the other hand, we identified 463, 1,066, and 55 differentially expressed proteins in the comparisons between PSP and HC, between PSP and PD, and between PD and HC, respectively, by the bootstrap ROC‐based statistical analysis. Although both analysis methods rendered differentially expressed proteins with *q*‐values < 0.05, the number of overlapping proteins between the two analytical methods were ∼48%, ∼68%, and ∼25% for PSP versus HC, PSP versus PD, and PD versus HC, respectively. The proteins with low *q*‐values were selected as differentially expressed proteins by both methods, while proteins with higher *q*‐values were selected by only one method (Data S3). These results suggest that the application of multiple statistical analysis methods can increase the confidence of selection for differentially expressed proteins.

In the gene set enrichment analysis, when PSP was compared to HC and PD, almost all proteins in the top five pathways were downregulated in PSP except for five proteins (SLC6A3, TH, UBE2L6, MAPT, and SOD2). Strikingly, most proteins enriched in the five pathways were mitochondrial proteins, and all of the mitochondrial proteins were downregulated except for SOD2. The upregulation of MAPT is expected since intracerebral accumulation of MAPT is a well‐known histopathologic feature of PSP.^41^, ^42^ SLC6A and TH, which were upregulated in PSP only when it was compared to PD, are proteins expressed in dopaminergic neurons.^43^, ^44^ We believe that these three proteins were identified from the dopaminergic neuronal axons projecting from substantia nigra to GP. The PPI analysis also demonstrated the clustering of mitochondrial proteins. When PSP was compared to HC, mitochondrial respiratory electron transport chain proteins and MRPs formed two main clusters. When PSP was compared to PD, only mitochondrial respiratory electron transport chain proteins formed the main cluster. Although the pathogenesis of PSP remains unclear, one of the known causes of parkinsonian disorders is neuronal cell death induced by inhibition of complex I in the mitochondrial respiratory electron chain.^45^, ^46^, ^47^, ^48^ We found many differentially expressed mitochondrial proteins included in complex I, such as NADH dehydrogenases (NDUFA4, NDUFA9, NDUFAF5, NDUFAF7, NDUFB11, NDUFB3, NDUFB4, NDUFB7, NDUFB8, NDUFC1, NDUFS2, NDUFS5, NDUFS7, NDUFS8, and so on) in this study. More strikingly, all the differentially expressed NADH dehydrogenase proteins were downregulated in PSP compared to both HC and PD. These proteins were included in all the top five pathways selected by the gene set enrichment analysis and formed the clusters in the PPI analysis. Other mitochondrial proteins such as complex II (succinate dehydrogenase), III (cytochrome b‐c1 complex), IV (cytochrome c oxidase), and V (ATP synthase) in the mitochondrial respiratory electron chain were also included in the differentially expressed proteins in the two comparisons between PSP versus HC and PD. When PSP was compared to HC, complex II, III, and IV proteins were enriched in all the top five pathways selected by the gene set enrichment analysis and complex III formed clusters by PPI analysis. When PSP was compared to PD, all of complex II, III, IV, and V were enriched in all of the top five pathways selected by the gene set enrichment analysis, and all of them also formed clusters in PPI analysis. Strikingly, all mitochondrial proteins related to complex I, II, III, IV, and V were downregulated in PSP compared to both HC and PD. This suggests that the mitochondrial dysfunction induced by the dysregulated mitochondrial respiratory electron transport chain complex could be a key component of the PSP pathogenesis accompanying MAPT aggregation. These results are in accordance with the previous reports that mitochondrial dysfunction is part of the etiopathogenesis of PSP.^49^

To analyse 45 samples, we conducted five batches of TMT experiments with the QC and the MP samples in each batch. Although the five batches of 11‐plex TMT‐based data were normalised by the MP samples, an obvious batch effect was observed, and further normalisation by the ComBat package removed it. This result suggests that simple normalisation by a common reference sample is often not enough to remove the batch effects when multiple batches of TMT experiments are conducted. Although we report that mitochondrial respiratory electron transport chain complex proteins were dysregulated in GP from PSP patients in this study, this outcome was derived from a mixture of multiple different cell types in GP. During the pathogenic process of neurodegenerative diseases, glial crosstalk is critical in the loss of cellular homeostasis and each cell type would show different responses to the inter‐ and intra‐cellular environment changes.^50^, ^51^, ^52^ Therefore, we need to deconvolute the proteome changes through cell‐type‐specific proteome analysis to understand the changes occurring in each cell type during the pathogenic process.

To the best of our knowledge, this is the first study focusing on proteomic analysis of GP from PSP patients. Our discovery of the link between dysregulated mitochondrial respiratory electron transport chain complex proteins and PSP provides a foundation for further investigation of PSP pathogenesis.

## CONFLICT OF INTEREST

We have no conflict of interest to declare.

## Supporting information

Supporting information
