# Correction to “Mass spectrometry‐based proteomics analysis of human globus pallidus from progressive supranuclear palsy patients discovers multiple disease pathways”

**DOI:** 10.1002/ctm2.1275

**Published:** 2023-05-22

Jang Y, Thuraisamy T, Redding‐Ochoa J, et al. Mass spectrometry‐based proteomics analysis of human globus pallidus from progressive supranuclear palsy patients discovers multiple disease pathways. *Clin Transl Med*. 2022;12:e1076. https://doi.org/10.1002/ctm2.1076

The sixth author's name and affiliation has been included.

The online article has been amended.

The authors apologize for this error.

