# Supplementary material for: Mass spectrometry‐based proteomics analysis of human globus pallidus from progressive supranuclear palsy patients discovers multiple disease pathways
Source: Clin Transl Med. 2022 Nov 10;12(11):e1076. doi: 10.1002/ctm2.1076 (PMC9647849; doi:10.1002/ctm2.1076)
Supplement: Supplementary file 1 — Supporting information [file CTM2-12-e1076-s001.docx]

Supplementary Materials

of

**Mass spectrometry-based proteomics analysis of human globus pallidus from** **progressive supranuclear palsy patients discovers multiple disease pathways**

Yura Jang^1,2^, Thujitha Thuraisamy^1,2^, Javier Redding-Ochoa^3^, Olga Pletnikova^3,8^, Juan C. Troncoso^2,3^, Liana S. Rosenthal^2^, Ted M. Dawson^1,2,4,5,6,7*^, Alexander Y. Pantelyat^2,*^, Chan Hyun Na^1,2,*^

^1^Neuroregeneration and Stem Cell Programs, Institute for Cell Engineering; ^2^Department of Neurology; ^3^Department of Pathology; ^4^Solomon H. Snyder Department of Neuroscience; ^5^Department of Pharmacology and Molecular Sciences, Johns Hopkins University School of Medicine, Baltimore, Maryland, USA; ^6^Adrienne Helis Malvin Medical Research Foundation; ^7^Diana Helis Henry Medical Research Foundation, New Orleans, LA, USA

^8^Current address: Department of Pathology and Anatomical Sciences, Jacobs School of Medicine and Biomedical Sciences, University at Buffalo, Buffalo, NY, USA

^*^To whom correspondence may be addressed. Email: chanhyun@jhmi.edu, tdawson@jhmi.edu, and apantel1@jhmi.edu

**Supplemental Table S1. Demographics of GP samples from PSP patients, HC individuals, and PD patients used in the Western blot experiment.**

| No. | Group | Age | Sex | Race | PMD (hours) | Diagnosis |
| --- | --- | --- | --- | --- | --- | --- |
| 1 | PSP | 80 | F | W | 5 | PSP |
| 2 | PSP | 73 | F | W | 18 | PSP |
| 3 | PSP | 77 | F | W | 8 | PSP |
| 4 | PSP | 73 | M | W | 8.5 | PSP |
| 5 | HC | 47 | M | W | 22 | Control |
| 6 | HC | 48 | F | B | 12 | Control |
| 7 | HC | 89 | F | W | 4 | Control |
| 8 | HC | 41 | M | W | 12 | Control |
| 9 | PD | 45 | F | W | 12 | PD |
| 10 | PD | 76 | M | W | 18 | PD |
| 11 | PD | 77 | M | W | 5 | PD dementia with Lewy bodies |
| 12 | PD | 75 | F | W | 24 | PD |

PSP: progressive supranuclear palsy, HC: healthy control, PD: Parkinson’s disease, M: Male, F: Female, W: White, B: Black, PMD: postmortem delay

**Supplemental Table S2. Differentially expressed proteins in PSP compared to HC calculated by SAM (top 50).** The number of differentially expressed proteins was 325.

| Protein name | Protein  symbol | *P* value | *q*-value | log2  (PSP/HC) |
| --- | --- | --- | --- | --- |
| Serum amyloid A-1 protein | SAA1 | 0.000365 | 0.006 | 3.453801 |
| Immunoglobulin heavy constant alpha 2 (Fragment) | IGHA2 | 0.000224 | 0.005333 | 3.256631 |
| Haptoglobin | HP | 0.000701 | 0.009581 | 2.936772 |
| Immunoglobulin heavy constant gamma 4 (Fragment) | IGHG4 | 0.000582 | 0.0104 | 2.906689 |
| Serum amyloid P-component | APCS | 0.000261 | 0.006261 | 2.858682 |
| Sequestosome-1 | SQSTM1 | 5.44E-07 | 0 | 2.800504 |
| Osteopontin | SPP1 | 2.07E-05 | 0.002667 | 2.62653 |
| Annexin A1 | ANXA1 | 0.000138 | 0.0056 | 2.506669 |
| Protein S100-A6 | S100A6 | 1.80E-05 | 0.002286 | 2.47084 |
| Tumor necrosis factor receptor superfamily member 16 | NGFR | 1.04E-05 | 0.0016 | 2.139886 |
| Transmembrane glycoprotein NMB | GPNMB | 2.58E-05 | 0.002571 | 1.984667 |
| Hemoglobin subunit delta | HBD | 0.000139 | 0.006714 | 1.966523 |
| Intercellular adhesion molecule 1 | ICAM1 | 2.35E-07 | 0 | 1.964058 |
| Chitinase-3-like protein 1 | CHI3L1 | 0.00034 | 0.009909 | 1.904309 |
| High mobility group protein HMG-I/HMG-Y | HMGA1 | 4.93E-09 | 0 | 1.760661 |
| Band 3 anion transport protein | SLC4A1 | 0.000121 | 0.008 | 1.743702 |
| EMILIN-1 | EMILIN1 | 5.43E-07 | 0.002769 | 1.077415 |
| Microtubule-associated protein | MAPT | 1.07E-05 | 0.008774 | 1.033958 |
| Methanethiol oxidase | SELENBP1 | 2.03E-05 | 0.009538 | 1.016508 |
| Phosphate carrier protein, mitochondrial | SLC25A3 | 1.60E-06 | 0.009838 | -0.78148 |
| Calcium-binding mitochondrial carrier protein Aralar1 | SLC25A12 | 9.94E-06 | 0.010692 | -0.8579 |
| ATPase family AAA domain-containing protein 1 | ATAD1 | 7.69E-07 | 0.005818 | -0.8769 |
| Heat shock 70 kDa protein 4L | HSPA4L | 9.95E-06 | 0.010261 | -0.88576 |
| Cytochrome c oxidase subunit 5A, mitochondrial | COX5A | 5.80E-06 | 0.009818 | -0.90936 |
| Mitochondrial pyruvate carrier 2 | MPC2 | 5.73E-06 | 0.00975 | -0.91182 |
| 28S ribosomal protein S2, mitochondrial | MRPS2 | 1.34E-05 | 0.0098 | -0.94704 |
| FAST kinase domain-containing protein 4 | TBRG4 | 1.97E-05 | 0.010128 | -0.96126 |
| [Pyruvate dehydrogenase (acetyl-transferring)] kinase isozyme 3, mitochondrial | PDK3 | 2.50E-05 | 0.010824 | -0.96186 |
| Leucine-rich PPR motif-containing protein, mitochondrial | LRPPRC | 5.30E-06 | 0.00637 | -1.01738 |
| Cytochrome b-c1 complex subunit 6, mitochondrial | UQCRH | 2.45E-05 | 0.009659 | -1.02504 |
| Cytochrome c oxidase subunit 1 | MT-CO1 | 7.74E-07 | 0.00275 | -1.03051 |
| Cytochrome c oxidase subunit 6B1 | COX6B1 | 6.48E-06 | 0.00624 | -1.05577 |
| Calcium-dependent secretion activator 1 | CADPS | 5.06E-05 | 0.009956 | -1.12664 |
| Cytochrome b-c1 complex subunit 8 | UQCRQ | 5.22E-05 | 0.009714 | -1.15426 |
| Small integral membrane protein 12 | SMIM12 | 2.16E-06 | 0.002667 | -1.16852 |
| LYR motif-containing protein 1 | LYRM1 | 4.27E-06 | 0.004706 | -1.19203 |
| SRA stem-loop-interacting RNA-binding protein, mitochondrial | SLIRP | 2.36E-06 | 0.003 | -1.28494 |
| Iron-sulfur cluster assembly enzyme ISCU, mitochondrial | ISCU | 2.43E-05 | 0.0065 | -1.29384 |
| 28S ribosomal protein S23, mitochondrial | MRPS23 | 7.62E-05 | 0.010111 | -1.29927 |
| Asparagine synthetase [glutamine-hydrolyzing] | ASNS | 9.15E-05 | 0.009579 | -1.34264 |
| Monocarboxylate transporter 4 | SLC16A3 | 0.00015 | 0.01 | -1.40099 |
| Kv channel-interacting protein 4 | KCNIP4 | 3.72E-05 | 0.005111 | -1.69721 |
| Calcium-dependent secretion activator 1 (Fragment) | CADPS | 7.81E-06 | 0.0024 | -1.70443 |
| FERM, ARHGEF and pleckstrin domain-containing protein 1 | FARP1 | 0.000208 | 0.010353 | -1.75516 |
| Mammalian ependymin-related protein 1 | EPDR1 | 7.02E-06 | 0.002222 | -1.76157 |
| Metabotropic glutamate receptor 1 | GRM1 | 5.01E-05 | 0.005684 | -1.78209 |
| Gamma-aminobutyric acid receptor subunit beta-2 | GABRB2 | 6.07E-06 | 0.002 | -1.7989 |
| Gamma-aminobutyric acid receptor subunit alpha-1 | GABRA1 | 1.65E-05 | 0.002909 | -1.87702 |
| Apolipoprotein A-IV | APOA4 | 0.000297 | 0.0084 | -2.47566 |
| Gap junction beta-6 protein | GJB6 | 1.65E-05 | 0.002 | -2.74853 |

**Supplemental Table S3.** **Differentially expressed proteins in PSP compared to PD calculated by SAM (top 50).** The number of differential proteins was 934.

| Protein name | Protein  symbol | *P* value | *q*-value | log2  (PSP/PD) |
| --- | --- | --- | --- | --- |
| Annexin A1 | ANXA1 | 2.12E-06 | 0 | 3.692431 |
| Sequestosome-1 | SQSTM1 | 1.13E-07 | 0 | 2.963463 |
| Protein S100-A6 | S100A6 | 1.30E-05 | 0.0004 | 2.712485 |
| Intercellular adhesion molecule 1 | ICAM1 | 1.89E-09 | 0 | 2.574009 |
| Complement C1q subcomponent subunit C | C1QC | 5.40E-07 | 0.000286 | 2.181405 |
| HLA class II histocompatibility antigen, DR alpha chain | HLA-DRA | 4.63E-06 | 0.000414 | 2.018529 |
| Argininosuccinate synthase | ASS1 | 8.69E-07 | 0.000222 | 1.992295 |
| Complement C1q subcomponent subunit B | C1QB | 1.49E-07 | 0 | 1.964902 |
| HLA class II histocompatibility antigen, DP alpha 1 chain | HLA-DPA1 | 5.71E-06 | 0.000353 | 1.95693 |
| Macrophage-capping protein | CAPG | 7.84E-06 | 0.000432 | 1.944502 |
| Thymidine phosphorylase | TYMP | 4.78E-06 | 0.000375 | 1.909451 |
| Protein S100-A11 | S100A11 | 5.26E-06 | 0.000343 | 1.810658 |
| High affinity immunoglobulin gamma Fc receptor I | FCGR1A | 1.23E-07 | 0 | 1.80381 |
| Guanylate-binding protein 1 | GBP1 | 1.72E-06 | 0.000429 | 1.673422 |
| Fructose-1,6-bisphosphatase 1 | FBP1 | 5.21E-07 | 0.000174 | 1.651545 |
| BAI1-associated protein 3 | BAIAP3 | 1.39E-05 | 0.000302 | 1.646963 |
| Matrilin-2 | MATN2 | 1.28E-06 | 0.000421 | 1.378534 |
| EMILIN-2 | EMILIN2 | 3.90E-06 | 0.00034 | 1.374375 |
| Glycogen phosphorylase, liver form | PYGL | 1.66E-06 | 0.00041 | 1.363667 |
| Fermitin family homolog 3 | FERMT3 | 6.09E-07 | 0.000444 | 1.277116 |
| Protein Niban | FAM129A | 1.92E-08 | 0.0002 | 1.221629 |
| Microtubule-associated protein | MAPT | 8.06E-07 | 0.00039 | 1.219158 |
| Long-chain fatty acid transport protein 3 | SLC27A3 | 1.49E-06 | 0.000348 | 1.210633 |
| Proteasome activator complex subunit 2 | PSME2 | 1.25E-08 | 0.00025 | 1.205257 |
| Tubulin-specific chaperone E | TBCE | 3.26E-09 | 0.000314 | -0.76631 |
| Band 4.1-like protein 3 | EPB41L3 | 1.52E-08 | 0.000308 | -0.82443 |
| COX assembly mitochondrial protein 2 homolog | CMC2 | 6.69E-08 | 0.000333 | -0.90891 |
| GTP-binding protein Di-Ras1 | DIRAS1 | 1.20E-07 | 0.0004 | -1.01764 |
| Cytochrome c oxidase subunit 5A, mitochondrial | COX5A | 2.96E-07 | 0.000327 | -1.01823 |
| 39S ribosomal protein L12, mitochondrial | MRPL12 | 2.34E-07 | 0.000364 | -1.02053 |
| Long-chain-fatty-acid--CoA ligase 6 | ACSL6 | 2.81E-09 | 0.000235 | -1.08398 |
| Calcium-binding mitochondrial carrier protein Aralar1 | SLC25A12 | 3.73E-10 | 0 | -1.1062 |
| Cytochrome c oxidase subunit 7A2, mitochondrial | COX7A2 | 7.40E-07 | 0.000356 | -1.1241 |
| Disintegrin and metalloproteinase domain-containing protein 22 | ADAM22 | 6.76E-09 | 0.000267 | -1.15783 |
| Disintegrin and metalloproteinase domain-containing protein 23 | ADAM23 | 9.31E-07 | 0.000381 | -1.18188 |
| Cytochrome c oxidase subunit 6B1 | COX6B1 | 2.05E-10 | 0 | -1.1929 |
| Cytochrome b-c1 complex subunit 8 | UQCRQ | 1.76E-07 | 0.000387 | -1.20965 |
| Cytochrome c oxidase subunit 1 | MT-CO1 | 3.09E-07 | 0.000364 | -1.24422 |
| BolA-like protein 3 | BOLA3 | 4.20E-06 | 0.000436 | -1.28574 |
| GTP-binding protein Di-Ras2 | DIRAS2 | 8.73E-08 | 0.00019 | -1.37235 |
| Cytochrome c oxidase subunit 6C | COX6C | 8.03E-08 | 0.000211 | -1.42113 |
| Potassium voltage-gated channel subfamily A member 1 | KCNA1 | 6.01E-06 | 0.000372 | -1.52167 |
| Hyaluronan and proteoglycan link protein 4 | HAPLN4 | 2.59E-07 | 0.000182 | -1.53117 |
| Adenylate cyclase type 1 | ADCY1 | 1.12E-05 | 0.00032 | -1.64437 |
| Calcium-dependent secretion activator 1 (Fragment) | CADPS | 3.69E-08 | 0 | -1.90665 |
| Gamma-aminobutyric acid receptor subunit beta-2 | GABRB2 | 1.21E-07 | 0 | -1.9092 |
| Gamma-aminobutyric acid receptor subunit alpha-1 | GABRA1 | 4.41E-08 | 0 | -2.03077 |
| Leucine-rich repeat LGI family member 2 | LGI2 | 4.05E-07 | 0 | -2.81795 |
| Gap junction beta-6 protein | GJB6 | 4.36E-08 | 0 | -3.23171 |
| Parvalbumin alpha | PVALB | 7.43E-07 | 0 | -3.95079 |

**Supplemental Table S4.** **Differentially expressed proteins in PD compared to HC calculated by SAM.** The number of differential proteins was 18.

| Protein name | Protein  symbol | *P* value | *q*-value | log2  (PD/HC) |
| --- | --- | --- | --- | --- |
| Kallikrein-6 | KLK6 | 4.56E-05 | 0.008 | 2.018432 |
| Sodium-coupled neutral amino acid transporter 2 | SLC38A2 | 0.000498 | 0.032 | 1.791496 |
| Selenoprotein P | SELENOP | 0.000285 | 0.031714 | 1.326592 |
| Activin receptor type-1 | ACVR1 | 5.32E-05 | 0.041176 | 0.858507 |
| Pyrroline-5-carboxylate reductase | PYCR3 | 1.00E-05 | 0.036267 | -0.74358 |
| Zinc finger and SCAN domain-containing protein 18 | ZSCAN18 | 3.87E-05 | 0.0212 | -1.11113 |
| High affinity immunoglobulin gamma Fc receptor I | FCGR1A | 0.000254 | 0.034 | -1.21786 |
| Glycogen phosphorylase, liver form | PYGL | 1.02E-05 | 0.008667 | -1.23285 |
| N-acetylglucosamine-1-phosphotransferase subunit gamma | GNPTG | 0.000251 | 0.033 | -1.33691 |
| Plexin-C1 | PLXNC1 | 0.000115 | 0.0195 | -1.5057 |
| SHC-transforming protein 3 | SHC3 | 0.000428 | 0.034154 | -1.51061 |
| Tryptophan 5-hydroxylase 2 | TPH2 | 4.02E-05 | 0.0072 | -1.78127 |
| High affinity immunoglobulin epsilon receptor subunit gamma | FCER1G | 0.000174 | 0.018857 | -1.86879 |
| Adipogenesis regulatory factor | ADIRF | 0.00038 | 0.023556 | -2.11576 |
| Protein FAM107A | FAM107A | 0.001839 | 0.0494 | -2.12398 |
| Aromatic-L-amino-acid decarboxylase | DDC | 6.18E-08 | 0 | -2.24654 |
| Tyrosine 3-monooxygenase | TH | 2.92E-07 | 0 | -3.50946 |
| Sodium-dependent dopamine transporter | SLC6A3 | 0.000113 | 0.001333 | -4.36124 |

**Supplemental Table S5.** **Differentially expressed proteins in PSP compared to HC calculated by bootstrap ROC analysis (top 50).** The number of differential proteins was 463.

| Protein name | Protein  symbol | *P* value | *q*-value | Mean | SD |
| --- | --- | --- | --- | --- | --- |
| High mobility group protein HMG-I/HMG-Y | HMGA1 | 5.16E-08 | 0 | 0.991111 | 0.011903 |
| Sequestosome-1 | SQSTM1 | 3.87E-07 | 0 | 0.973333 | 0.022775 |
| EMILIN-1 | EMILIN1 | 1.25E-06 | 0 | 0.96 | 0.030803 |
| Intercellular adhesion molecule 1 | ICAM1 | 1.25E-06 | 0 | 0.96 | 0.031765 |
| Ras suppressor protein 1 | RSU1 | 3.51E-06 | 0 | 0.946667 | 0.037769 |
| U6 snRNA-associated Sm-like protein LSm8 | LSM8 | 8.82E-06 | 0 | 0.933333 | 0.042797 |
| REST corepressor 3 | RCOR3 | 1.18E-05 | 0 | 0.928889 | 0.045818 |
| U6 snRNA-associated Sm-like protein LSm4 (Fragment) | LSM4 | 2.04E-05 | 0 | 0.92 | 0.05024 |
| Zinc finger CCCH domain-containing protein 18 | ZC3H18 | 3.43E-05 | 0 | 0.911111 | 0.0503 |
| Microtubule-associated protein | MAPT_ | 3.43E-05 | 0 | 0.911111 | 0.053284 |
| RNA-binding protein 8A | RBM8A | 7.12E-05 | 0 | 0.897778 | 0.052203 |
| Osteopontin | SPP1 | 7.12E-05 | 0 | 0.897778 | 0.055128 |
| Glycophorin-C | GYPC | 8.98E-05 | 0 | 0.893333 | 0.055744 |
| Cytochrome b-c1 complex subunit 9 | UQCR10 | 7.12E-05 | 0 | 0.102222 | 0.053829 |
| GTP-binding protein Di-Ras1 | DIRAS1 | 7.12E-05 | 0 | 0.102222 | 0.055907 |
| Metabotropic glutamate receptor 1 | GRM1 | 7.12E-05 | 0 | 0.102222 | 0.056588 |
| Mitochondrial dicarboxylate carrier | SLC25A10 | 5.62E-05 | 0 | 0.097778 | 0.052463 |
| Metaxin-2 | MTX2 | 4.40E-05 | 0 | 0.093333 | 0.051761 |
| Cleft lip and palate transmembrane protein 1 | CLPTM1 | 4.40E-05 | 0 | 0.093333 | 0.054165 |
| Cytochrome b-c1 complex subunit 6, mitochondrial | UQCRH | 4.40E-05 | 0 | 0.093333 | 0.056681 |
| COX assembly mitochondrial protein 2 homolog | CMC2 | 3.43E-05 | 0 | 0.088889 | 0.048709 |
| 28S ribosomal protein S35, mitochondrial | MRPS35 | 3.43E-05 | 0 | 0.088889 | 0.049518 |
| Calcium-dependent secretion activator 1 | CADPS | 3.43E-05 | 0 | 0.088889 | 0.054026 |
| Peptidyl-tRNA hydrolase ICT1, mitochondrial | MRPL58 | 3.43E-05 | 0 | 0.088889 | 0.054391 |
| Kv channel-interacting protein 4 | KCNIP4 | 3.43E-05 | 0 | 0.088889 | 0.055634 |
| 28S ribosomal protein S9, mitochondrial | MRPS9 | 2.65E-05 | 0 | 0.084444 | 0.048686 |
| 39S ribosomal protein L9, mitochondrial | MRPL9 | 2.65E-05 | 0 | 0.084444 | 0.04947 |
| Iron-sulfur cluster assembly enzyme ISCU, mitochondrial | ISCU | 2.65E-05 | 0 | 0.084444 | 0.049742 |
| Gamma-aminobutyric acid receptor subunit alpha-1 | GABRA1 | 2.65E-05 | 0 | 0.084444 | 0.050297 |
| SRA stem-loop-interacting RNA-binding protein, mitochondrial | SLIRP | 2.04E-05 | 0 | 0.08 | 0.04925 |
| Heat shock 70 kDa protein 4L | HSPA4L | 2.04E-05 | 0 | 0.08 | 0.049324 |
| Elongation factor G, mitochondrial | GFM1 | 2.04E-05 | 0 | 0.08 | 0.050098 |
| FAST kinase domain-containing protein 4 | TBRG4 | 2.04E-05 | 0 | 0.08 | 0.053026 |
| Enoyl-[acyl-carrier-protein] reductase, mitochondrial | MECR | 2.04E-05 | 0 | 0.08 | 0.053948 |
| 39S ribosomal protein L17, mitochondrial | MRPL17 | 2.04E-05 | 0 | 0.08 | 0.054324 |
| Gamma-glutamyl hydrolase | GGH | 2.04E-05 | 0 | 0.08 | 0.054724 |
| 39S ribosomal protein L16, mitochondrial | MRPL16 | 2.04E-05 | 0 | 0.08 | 0.055641 |
| LYR motif-containing protein 1 | LYRM1 | 1.55E-05 | 0 | 0.075556 | 0.046289 |
| 28S ribosomal protein S2, mitochondrial | MRPS2 | 1.55E-05 | 0 | 0.075556 | 0.048077 |
| Calcium-dependent secretion activator 1 (Fragment) | CADPS | 1.55E-05 | 0 | 0.075556 | 0.051456 |
| Gamma-aminobutyric acid receptor subunit beta-2 | GABRB2 | 1.18E-05 | 0 | 0.071111 | 0.043391 |
| Phosphate carrier protein, mitochondrial | SLC25A3 | 1.18E-05 | 0 | 0.071111 | 0.047329 |
| 39S ribosomal protein L12, mitochondrial | MRPL12 | 1.18E-05 | 0 | 0.071111 | 0.047434 |
| Leucine-rich PPR motif-containing protein, mitochondrial | LRPPRC | 8.82E-06 | 0 | 0.066667 | 0.043447 |
| Mitochondrial pyruvate carrier 2 | MPC2 | 8.82E-06 | 0 | 0.066667 | 0.044396 |
| Cytochrome c oxidase subunit 5A, mitochondrial | COX5A | 8.82E-06 | 0 | 0.066667 | 0.050933 |
| Small integral membrane protein 12 | SMIM12 | 4.81E-06 | 0 | 0.057778 | 0.049675 |
| Mammalian ependymin-related protein 1 | EPDR1 | 2.51E-06 | 0 | 0.048889 | 0.048204 |
| ATPase family AAA domain-containing protein 1 | ATAD1 | 1.79E-06 | 0 | 0.044444 | 0.032361 |
| Cytochrome c oxidase subunit 1 | MT-CO1 | 1.25E-06 | 0 | 0.04 | 0.030657 |

**Supplemental Table S6.** **Differentially expressed proteins in PSP compared to PD calculated by bootstrap ROC analysis (top 50).** The number of differential proteins was 1,066.

| Protein name | Protein  symbol | *P* value | *q*-value | Mean | SD |
| --- | --- | --- | --- | --- | --- |
| Fermitin family homolog 3 | FERMT3 | 2.58E-08 | 0 | 0.995556 | 0.007543 |
| High affinity immunoglobulin gamma Fc receptor I | FCGR1A | 9.03E-08 | 0 | 0.986667 | 0.015066 |
| Sequestosome-1 | SQSTM1 | 9.03E-08 | 0 | 0.986667 | 0.015384 |
| Intercellular adhesion molecule 1 | ICAM1 | 1.55E-07 | 0 | 0.982222 | 0.019863 |
| Serine/threonine-protein kinase 4 | STK4 | 2.45E-07 | 0 | 0.977778 | 0.02492 |
| Poly [ADP-ribose] polymerase 9 | PARP9 | 3.87E-07 | 0 | 0.973333 | 0.026458 |
| Proteasome activator complex subunit 2 | PSME2 | 3.87E-07 | 0 | 0.973333 | 0.027687 |
| Protein S100-A11 | S100A11 | 5.80E-07 | 0 | 0.968889 | 0.030027 |
| Argininosuccinate synthase | ASS1 | 8.64E-07 | 0 | 0.964444 | 0.030566 |
| Complement C1q subcomponent subunit B | C1QB | 1.25E-06 | 0 | 0.96 | 0.031151 |
| U6 snRNA-associated Sm-like protein LSm4 (Fragment) | LSM4 | 1.79E-06 | 0 | 0.955556 | 0.033657 |
| Fructose-1,6-bisphosphatase 1 | FBP1 | 1.79E-06 | 0 | 0.955556 | 0.034032 |
| Complement C1q subcomponent subunit C | C1QC | 1.79E-06 | 0 | 0.955556 | 0.035076 |
| Lamin-B1 | LMNB1 | 2.51E-06 | 0 | 0.951111 | 0.036432 |
| Matrilin-2 | MATN2 | 3.51E-06 | 0 | 0.946667 | 0.038778 |
| Long-chain fatty acid transport protein 3 | SLC27A3 | 3.51E-06 | 0 | 0.946667 | 0.038863 |
| Leucine-rich repeat LGI family member 2 | LGI2 | 4.81E-06 | 0 | 0.057778 | 0.038114 |
| COX assembly mitochondrial protein homolog | CMC1 | 3.51E-06 | 0 | 0.053333 | 0.039072 |
| Uncharacterized protein C6orf136 (Fragment) | C6orf136 | 2.51E-06 | 0 | 0.048889 | 0.034395 |
| Sodium- and chloride-dependent creatine transporter 1 | SLC6A8 | 2.51E-06 | 0 | 0.048889 | 0.035743 |
| Uncharacterized protein (Accession_U3KQK5) | - | 2.51E-06 | 0 | 0.048889 | 0.036555 |
| ATPase family AAA domain-containing protein 1 | ATAD1 | 1.79E-06 | 0 | 0.044444 | 0.032782 |
| Solute carrier family 25 member 40 | SLC25A40 | 1.79E-06 | 0 | 0.044444 | 0.034305 |
| Cytochrome c oxidase subunit 7A2, mitochondrial | COX7A2 | 1.79E-06 | 0 | 0.044444 | 0.035721 |
| Cytoglobin | CYGB | 1.79E-06 | 0 | 0.044444 | 0.037586 |
| Rap1 GTPase-GDP dissociation stimulator 1 | RAP1GDS1 | 1.25E-06 | 0 | 0.04 | 0.031208 |
| ADP/ATP translocase 2 | SLC25A5 | 1.25E-06 | 0 | 0.04 | 0.031519 |
| Cytochrome c oxidase subunit 1 | MT-CO1 | 8.64E-07 | 0 | 0.035556 | 0.028083 |
| Cytochrome b-c1 complex subunit 1, mitochondrial | UQCRC1 | 8.64E-07 | 0 | 0.035556 | 0.028283 |
| Cytochrome b-c1 complex subunit 8 | UQCRQ | 8.64E-07 | 0 | 0.035556 | 0.028453 |
| Hyaluronan and proteoglycan link protein 4 | HAPLN4 | 8.64E-07 | 0 | 0.035556 | 0.029558 |
| Gamma-aminobutyric acid receptor subunit beta-2 | GABRB2 | 8.64E-07 | 0 | 0.035556 | 0.031321 |
| Mitochondrial import inner membrane translocase subunit Tim13 | TIMM13 | 8.64E-07 | 0 | 0.035556 | 0.031417 |
| ATP synthase mitochondrial F1 complex assembly factor 1 | ATPAF1 | 8.64E-07 | 0 | 0.035556 | 0.034742 |
| 39S ribosomal protein L12, mitochondrial | MRPL12 | 8.64E-07 | 0 | 0.035556 | 0.037649 |
| Gamma-aminobutyric acid receptor subunit alpha-1 | GABRA1 | 5.80E-07 | 0 | 0.031111 | 0.027118 |
| Mitochondrial carrier homolog 2 | MTCH2 | 5.80E-07 | 0 | 0.031111 | 0.028798 |
| Tubulin-specific chaperone E | TBCE | 5.80E-07 | 0 | 0.031111 | 0.029127 |
| Mitochondrial pyruvate carrier 2 | MPC2 | 3.87E-07 | 0 | 0.026667 | 0.023606 |
| Cytochrome c oxidase subunit 6C | COX6C | 3.87E-07 | 0 | 0.026667 | 0.024286 |
| GTP-binding protein Di-Ras2 | DIRAS2 | 3.87E-07 | 0 | 0.026667 | 0.024832 |
| GTP-binding protein Di-Ras1 | DIRAS1 | 3.87E-07 | 0 | 0.026667 | 0.025711 |
| Disintegrin and metalloproteinase domain-containing protein 22 | ADAM22 | 3.87E-07 | 0 | 0.026667 | 0.026698 |
| Calcium-dependent secretion activator 1 (Fragment) | CADPS | 1.55E-07 | 0 | 0.017778 | 0.020082 |
| Gap junction beta-6 protein | GJB6 | 1.55E-07 | 0 | 0.017778 | 0.020507 |
| Cytochrome c oxidase subunit 6B1 | COX6B1 | 9.03E-08 | 0 | 0.013333 | 0.014175 |
| AFG1-like ATPase | AFG1L | 9.03E-08 | 0 | 0.013333 | 0.016863 |
| COX assembly mitochondrial protein 2 homolog | CMC2 | 2.58E-08 | 0 | 0.004444 | 0.007816 |
| Long-chain-fatty-acid--CoA ligase 6 | ACSL6 | 1.29E-08 | 0 | 0 | 0 |
| Calcium-binding mitochondrial carrier protein Aralar1 | SLC25A12 | 1.29E-08 | 0 | 0 | 0 |

**Supplemental Table S7.** **Differentially expressed proteins in PD compared to HC calculated by bootstrap ROC analysis (top 50).** The number of differential proteins was 55.

| Protein name | Protein  symbol | *P* value | *q*-value | Mean | SD |
| --- | --- | --- | --- | --- | --- |
| Adenylosuccinate lyase | ADSL | 2.04E-05 | 0 | 0.92 | 0.05007 |
| Proteasome subunit beta type-2 | PSMB2 | 4.40E-05 | 0 | 0.906667 | 0.058698 |
| FAD synthase | FLAD1 | 5.62E-05 | 0 | 0.902222 | 0.055438 |
| GTP-binding protein SAR1b | SAR1B | 5.62E-05 | 0 | 0.902222 | 0.058283 |
| WD repeat and FYVE domain-containing protein 2 | WDFY2 | 8.98E-05 | 0 | 0.893333 | 0.059058 |
| Phosphatidylinositol glycan anchor biosynthesis class U protein | PIGU | 0.000113 | 0 | 0.888889 | 0.057852 |
| Activin receptor type-1 | ACVR1 | 0.00014 | 0 | 0.884444 | 0.059958 |
| Proteasome subunit beta type-5 | PSMB5 | 0.00014 | 0 | 0.884444 | 0.060517 |
| Sodium-coupled neutral amino acid transporter 2 | SLC38A2 | 0.000174 | 0 | 0.88 | 0.065532 |
| Kallikrein-6 | KLK6 | 0.000174 | 0 | 0.88 | 0.065975 |
| Sestrin-1 | SESN1 | 0.000215 | 0 | 0.875556 | 0.061169 |
| Tubulin-specific chaperone E | TBCE | 0.000215 | 0 | 0.875556 | 0.063114 |
| Multiple epidermal growth factor-like domains protein 10 | MEGF10 | 0.000264 | 0 | 0.871111 | 0.066756 |
| Mitochondrial carrier homolog 2 | MTCH2 | 0.000323 | 0 | 0.866667 | 0.064123 |
| Cysteine conjugate-beta lyase cytoplasmic (Glutamine transaminase K, kyneurenine aminotransferase), isoform CRA_b | KYAT1 | 0.000323 | 0 | 0.866667 | 0.064689 |
| ATP-dependent RNA helicase DDX39A | DDX39A | 0.000323 | 0 | 0.866667 | 0.067641 |
| GTP-binding protein Rit1 | RIT1 | 0.000323 | 0 | 0.866667 | 0.068459 |
| Coiled-coil domain-containing protein 47 | CCDC47 | 0.000394 | 0 | 0.862222 | 0.06464 |
| Selenoprotein P | SELENOP | 0.000394 | 0 | 0.862222 | 0.067355 |
| Non-homologous end-joining factor 1 | NHEJ1 | 0.000394 | 0 | 0.862222 | 0.069741 |
| Ras-related protein Rab-8A | RAB8A | 0.000478 | 0 | 0.857778 | 0.072511 |
| Ribonuclease inhibitor | RNH1 | 0.000478 | 0 | 0.857778 | 0.073579 |
| N-acylethanolamine-hydrolyzing acid amidase | NAAA | 0.000478 | 0 | 0.857778 | 0.076299 |
| Tyrosine--tRNA ligase, cytoplasmic | YARS | 0.000696 | 0.030303 | 0.848889 | 0.071945 |
| Transforming acidic coiled-coil-containing protein 1 (Fragment) | TACC1 | 0.000696 | 0.03125 | 0.848889 | 0.070108 |
| Testican-3 | SPOCK3 | 0.000696 | 0.032258 | 0.848889 | 0.071169 |
| Peptidylprolyl isomerase domain and WD repeat-containing protein 1 | PPWD1 | 0.000696 | 0.033333 | 0.848889 | 0.075376 |
| Phosphoglycerate mutase 1 | PGAM1 | 0.000696 | 0.034483 | 0.848889 | 0.069823 |
| Sodium-dependent serotonin transporter | SLC6A4 | 0.000578 | 0 | 0.146667 | 0.068141 |
| High affinity immunoglobulin epsilon receptor subunit gamma | FCER1G | 0.000578 | 0 | 0.146667 | 0.074274 |
| Superoxide dismutase [Mn], mitochondrial | SOD2 | 0.000578 | 0 | 0.146667 | 0.079706 |
| SHC-transforming protein 3 | SHC3 | 0.000478 | 0 | 0.142222 | 0.068696 |
| Mesoderm-specific transcript homolog protein | MEST | 0.000478 | 0 | 0.142222 | 0.073062 |
| Uncharacterized protein KIAA1671 | KIAA1671 | 0.000394 | 0 | 0.137778 | 0.069895 |
| Serine/threonine-protein kinase Nek9 | NEK9 | 0.000394 | 0 | 0.137778 | 0.069977 |
| Golgin subfamily A member 3 | GOLGA3 | 0.000264 | 0 | 0.128889 | 0.064794 |
| Ubiquitin carboxyl-terminal hydrolase 11 | USP11 | 0.000264 | 0 | 0.128889 | 0.066158 |
| IgGFc-binding protein | FCGBP | 0.000264 | 0 | 0.128889 | 0.068731 |
| Inositol polyphosphate 5-phosphatase OCRL-1 | OCRL | 0.000215 | 0 | 0.124444 | 0.062789 |
| N-acetylglucosamine-1-phosphotransferase subunit gamma | GNPTG | 0.000215 | 0 | 0.124444 | 0.064675 |
| Sodium-dependent dopamine transporter | SLC6A3 | 0.000174 | 0 | 0.12 | 0.061889 |
| 40S ribosomal protein S10 | RPS10 | 0.00014 | 0 | 0.115556 | 0.064175 |
| Zinc finger and SCAN domain-containing protein 18 | ZSCAN18 | 5.62E-05 | 0 | 0.097778 | 0.060878 |
| Serine/threonine-protein kinase WNK2 | WNK2 | 3.43E-05 | 0 | 0.088889 | 0.052743 |
| Plexin-C1 | PLXNC1 | 2.04E-05 | 0 | 0.08 | 0.054103 |
| Tryptophan 5-hydroxylase 2 | TPH2 | 1.55E-05 | 0 | 0.075556 | 0.044471 |
| Pyrroline-5-carboxylate reductase | PYCR3 | 1.55E-05 | 0 | 0.075556 | 0.048983 |
| Glycogen phosphorylase, liver form | PYGL | 8.82E-06 | 0 | 0.066667 | 0.043605 |
| Tyrosine 3-monooxygenase | TH | 2.51E-06 | 0 | 0.048889 | 0.043824 |
| Aromatic-L-amino-acid decarboxylase | DDC | 5.16E-08 | 0 | 0.008889 | 0.011988 |

**Supplemental Table S8. Pathways enriched by gene set enrichment pathways of the M12 and the M11 modules generated WGCNA of PSP and HC.**

| Term | Count | *PH | % | *P* value |
| --- | --- | --- | --- | --- |
| M12 |  |  |  |  |
| Parkinson's disease | 55 | 142 | 38.7 | 3.40E-48 |
| Oxidative phosphorylation | 51 | 133 | 38.3 | 3.20E-44 |
| Huntington's disease | 56 | 192 | 29.2 | 2.20E-41 |
| Alzheimer's disease | 51 | 168 | 30.4 | 2.10E-38 |
| Non-alcoholic fatty liver disease (NAFLD) | 48 | 151 | 31.8 | 4.70E-37 |
| Metabolic pathways | 99 | 1219 | 8.1 | 3.70E-25 |
| Carbon metabolism | 26 | 113 | 23.0 | 7.00E-16 |
| Citrate cycle (TCA cycle) | 14 | 30 | 46.7 | 5.20E-13 |
| Biosynthesis of antibiotics | 25 | 212 | 11.8 | 7.40E-09 |
| Cardiac muscle contraction | 15 | 75 | 20.0 | 2.20E-08 |
| M11 |  |  |  |  |
| Ribosome | 25 | 136 | 18.4 | 5.50E-24 |
| Aminoacyl-tRNA biosynthesis | 4 | 66 | 6.1 | 3.10E-02 |
| Central carbon metabolism in cancer | 3 | 64 | 4.7 | 1.40E-01 |
| Thyroid hormone synthesis | 3 | 70 | 4.3 | 1.70E-01 |
| Protein digestion and absorption | 3 | 88 | 3.4 | 2.30E-01 |

*PH: the total number of proteins in the pathway

**Supplemental Table S9. Pathways enriched ty gene set enriched pathways of the M6 module generated by WGCNA of PSP and PD.**

| Term | Count | *PH | % | *P* value |
| --- | --- | --- | --- | --- |
| Parkinson's disease | 54 | 142 | 38.0 | 1.00E-25 |
| Oxidative phosphorylation | 51 | 133 | 38.3 | 1.80E-24 |
| Huntington's disease | 56 | 192 | 29.2 | 2.60E-20 |
| Alzheimer's disease | 52 | 168 | 31.0 | 4.00E-20 |
| Non-alcoholic fatty liver disease (NAFLD) | 49 | 151 | 32.5 | 6.30E-20 |
| Metabolic pathways | 143 | 1219 | 11.7 | 1.30E-10 |
| Spliceosome | 26 | 133 | 19.5 | 5.70E-06 |
| Cardiac muscle contraction | 18 | 75 | 24.0 | 1.50E-05 |
| RNA transport | 28 | 172 | 16.3 | 7.30E-05 |
| Carbon metabolism | 21 | 113 | 18.6 | 1.20E-04 |

*PH: the total number of proteins in the pathway


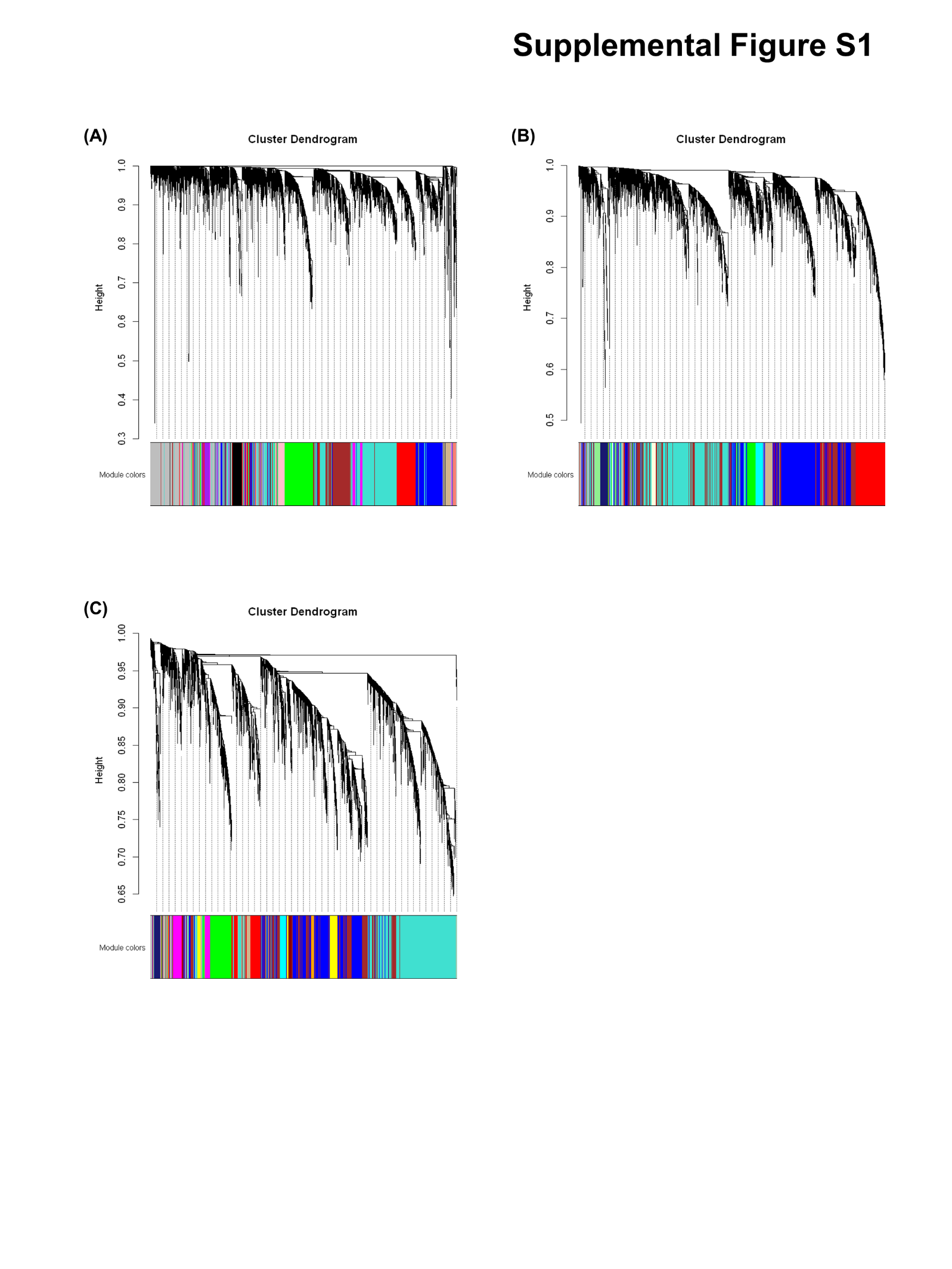


**Supplemental Figure S1. Cluster dendrogram of WGCNA.**

Cluster dendrogram created by WGCNA of (A) PSP and HC, (B) PSP and PD, and (C) PD and HC to identify protein modules that show co-expression patterns.

**
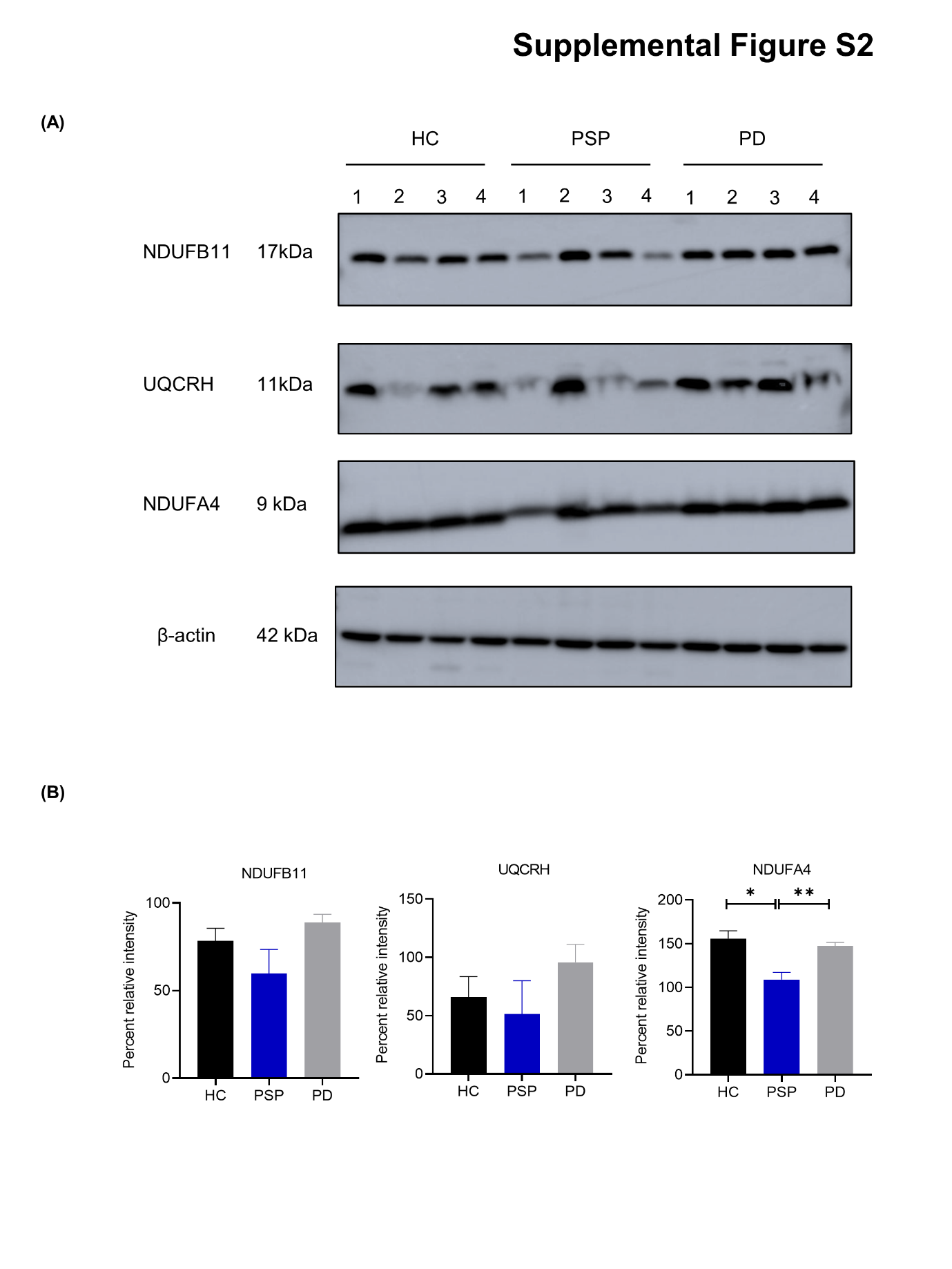
**

**Supplemental Figure S2. Validation of selected proteins in mitochondrial electron transport chain in the GP of HC, PSP, and PD samples using Western blot**.

(A) Western blot experiments for NDUFB11, UQCRH, and NDUFA4 using the GP from PSP, PD, and HC individuals were conducted. (B) Quantification of NDUFB11, UQCRH, and NDUFA4 relative to *β*-actin was conducted using ImageJ. Data in the graphs indicate mean ± SEM (*t*-test, **P* < 0.05, ***P* < 0.001, *n* = 4 independent samples).

**Supplemental Data S1. List of proteins identified in this study**

**Supplemental Data S2. List of quantified proteins including statistical analysis results**

**Supplemental Data S3. List of proteins enriched in the gene set enrichment analysis**

**Supplemental Data S4. List of proteins in the WGCNA modules**

**FUNDING**

This work was supported by an NIH grant (U01 NS102035 to A.Y. P and T.M.D.).
